# The Effect of Green Extraction Technologies on the Chemical Composition of Medicinal Chaga Mushroom Extracts

**DOI:** 10.3390/jof10030225

**Published:** 2024-03-19

**Authors:** Vesna Lazić, Anita Klaus, Maja Kozarski, Ana Doroški, Tomislav Tosti, Siniša Simić, Jovana Vunduk

**Affiliations:** 1 Institute for Food Technology and Biochemistry, Faculty of Agriculture, University of Belgrade, Nemanjina 6, 11080 Belgrade, Serbia; vlazic93@gmail.com (V.L.); aklaus@agrif.bg.ac.rs (A.K.); maja@agrif.bg.ac.rs (M.K.); ana.doroski@agrif.bg.ac.rs (A.D.); 2 Institute of Chemistry, Technology and Metallurgy-National Institute of the Republic of Serbia, University of Belgrade, Studentski Trg 12–16, 11158 Belgrade, Serbia; tosti@chem.bg.ac.rs; 3 Faculty of Technology, University of Novi Sad, Bulevar Cara Lazara 1, 21000 Novi Sad, Serbia; sinisa.simic@uns.ac.rs; 4 Institute of General and Physical Chemistry, Studentski Trg 12/V, 11158 Belgrade, Serbia

**Keywords:** medicinal mushroom, green extraction, *Inonotus obliquus*, microwave-assisted extraction, ultrasonic-assisted extraction, subcritical water extraction

## Abstract

The mushroom industry should implement green extraction technologies; however, there is not enough information on the differences between these techniques expressed as the chemical composition of the resulting extract. In this study, selected types of green extraction techniques (GETs) were used on Chaga (*Inonotus obliquus*) (Fr.) Pilát from Serbia (IS) and Mongolia (IM) to examine the differences that would enable the composition-based technology choices in the mushroom supplement industry. Subcritical water extraction (SWE), microwave-assisted (MW) extraction, and ultrasonic-assisted extraction (VAE) were used to prepare the extracts. SWE was performed at two different temperatures (120 and 200 °C), while 96% ethanol, 50% ethanol, and water were used for MW and VAE. The yield, the content of total phenols, total proteins, and carbohydrates, qualitative and quantitative analysis of phenolic compounds, carbohydrates, including α- and β- and total glucans, and fatty acids, were determined in the obtained extracts. SWE resulted in a significantly higher yield, total polysaccharide, and glucan content than any other technique. Glucose was the most dominant monosaccharide in the SWE samples, especially those extracted at 200 °C. The MW 50% EtOH extracts showed the highest yield of total phenols. Among the tested phenolic compounds, chlorogenic acid was the most dominant. SWE can be recommended as the most efficient method for extracting commercially important compounds, especially glucans and phenols.

## 1. Introduction

The last decade was marked by a tremendous increase in the use of medicinal mushrooms as dietary supplements, adjuvants, and functional food [1]. This millennia-old source of remedies is experiencing a renaissance heavily supported by the development of technology. The high ratio of manual work, especially in raw material production, decreased due to the introduction of mechanical solutions (e.g., automated trolleys for mushroom picking) fed by fossil fuels. Further processing includes extractions, the use of chemicals, and processing technology. Down the line, we produce faster, more, purer, and ever more efficient. However, there is a cost in gaseous form, the burden on Earth’s shoulders which is the carbon footprint. Commercially used medicinal mushrooms are cultivated thus as a part of agriculture, forestry, and land use with a carbon emission of 18.4% [2]; they are processed into the final products counting as chemical and petrochemical production (including pharmaceuticals) with 3.6% of global greenhouse gas emission. Both phases heavily rely on fossil fuels either to run production or to manufacture chemicals (e.g., solvents) causing greenhouse gas emissions of almost 200 million tons per year, with an increase of 10% from 2012 to 2021, as reported by the United States Environmental Protection Agency [3]. So, how can we achieve the Sustainable Development Goals (SDGs) or Paris Agreement and approach net zero emissions by 2050 [4]? We have already passed the first base, the recognition of the problem and its complexity. The next, which is to tackle the problem from every single angle, is also in progress and strongly supported by the research community [5]. Green technologies (GTs), used to manufacture goods and services with a smaller or zero carbon footprint, represent a growing opportunity to decarbonize among others, chemistry, medicine, agriculture, and food sectors. As discussed by Bradu et al. [5] there are several options to achieve the SDGs, e.g., developing environmental biotechnology, using bio-based materials and bioenergy, engineering and producing chemicals in a greener way, and these should all be supported by proper legislation. The chemicals and pharmaceutical products we use daily are costly for the environment. As reported by the European Commission, every kg of active ingredient takes about 100 kg of different materials to produce [6]. On one hand, we use natural products and dietary supplements due to their health benefits, natural origin, and fewer or no side effects while on the other hand, we increase our planet’s vulnerability [7]. For example, polysaccharide extracts derived from medicinal mushrooms are commercially produced by a combination of ethanol and high-pressure water extraction which is time, labor, and energy-consuming [8]. In addition, some of these conventional techniques require toxic organic solvents, give low yields, can cause thermal degradation of active compounds, loss of volatile compounds, have low extraction selectivity, and can lead to the presence of residues in the desired compounds and final products [9,10,11]. All this has led to an interest in new extraction methods that will be ecologically and economically acceptable [12,13] and are merged under the term Green Extraction Technologies (GETs). They include ultrasound-assisted extraction, microwave-assisted extraction, high-pressure, subcritical, and supercritical water extraction, pressurized liquid extraction, negative pressure cavitations-assisted extraction, enzyme-assisted extraction, pulsed electric field-assisted extraction, and accelerated solvent extraction. The goal is to limit, lower, or exclude the use of solvent, minimize the use of energy, generate fewer by-products and waste streams, shorten the extraction time, and improve natural ingredient recovery [14].

Companies dealing with medicinal mushroom processing are already adopting the GT approach and developing products based on supercritical extraction (e.g., mushroom spore oil) or promoting proprietary processes characterized by fewer chemicals or solvent-free extraction [15]. Calleja-Gómez et al. [16] applied a pulsed electric field-assisted extraction for the recovery of nutrients and bioactive compounds from edible *Agaricus bisporus* mushroom and evaluated the best conditions to reduce the use of organic solvents. The authors reported increased recovery of phenolic compounds, carbohydrates, proteins, and minerals when compared with conventional extraction with shaking. Similarly, Mishra et al. [10] optimized the supercritical CO_2_ extraction parameters to obtain more bioactive *Ophiocordyceps sinensis* (*Cordyceps sinensis* in the reference paper) extracts. This technique proved to be protective of mushrooms’ thermolabile compounds. It has been stated that CO_2_ is generally a solvent of preference in industry. It is easy to remove, has better diffusion, and has lower temperature requirements. In another study, oyster mushroom (*Pleurotus ostreatus*) was treated with hot water and supercritical CO_2_ to obtain extracts rich in antioxidant polysaccharides [17]. Several medicinal mushroom species have been extracted by the subcritical water technique as an environmentally friendly method while giving biologically active products [18]. When compared with commercial products, these extracts had satisfactory chemical composition and enhanced physical properties when incorporated into advanced products like hydrogels. However, the comparison of several green technologies and their effect on chemical composition has not been performed on mushroom material. Although they are all marked as green, each one has specific benefits, and the final extracts differ in chemical and thus biological aspects.

Hereby, we compared several extraction techniques referred to as GETs by examining the chemical profile, including the extract yield and qualitative and quantitative analysis of industrially important classes of biological compounds, of the commercially important medicinal mushroom *I. obliquus* from two different sources. The results presented should enable researchers and the mushroom and nutraceutical industry to select optimal GETs relevant to the aim of the application.

## 2. Materials and Methods

### 2.1. Reagents and Standards

Gallic acid, phenol, Brilliant Blue G, Folin–Ciocalteu reagent, and albumin, were purchased from Sigma-Aldrich (St. Louis, MO, USA). D-(+)-glucose, dimethylsulfoxide (DMSO), and ethanol was obtained from Fisher Scientific, (Loughborough, UK). Hydrochloric acid (HCl) was purchased from Zorka Pharma (Šabac, Serbia). A Mushroom and Yeast β-glucan Assay K-YBGL09/2009 (Megazyme Int., Wicklow, Ireland) was used. Deionized water was used in all experiments unless stated otherwise. The sugar standards were as follows: glucose, fructose, saccharose, trehalose, turanose, galactose, ribose, maltose, and arabinose were purchased from TCI Europe N.V. (Zwijndrecht, Belgium), turanose, rhamnose, isomaltose, panose, raffinose, isomaltotriose, maltotriose, melibiose, xylose, melesitose, and stachyose were obtained from Tokyo Chemical Industry (TCI, Tokyo, Japan). Sorbitol, erythritol (Ert), arabinitol (Arabt), mannitol (Mant), and galactitol were purchased from Sigma-Aldrich, Merck (Steinheim, Germany). Ultra-pure water (MicroPure water purification system, 0.055 μS/cm, TKA, Thermo Fisher Scientific, Niederelbert, Germany) was used to prepare standard sugar solutions and blanks. Methanol, chloroform, diethyl ether, acetone, and n-hexane were from Merck (KGaA, Darmstadt, Germany). Syringe filters (13 mm, polytetrafluoroethylene (PTFE) membrane 0.45 μm) were purchased from Supelco (Bellefonte, PA, USA). Phenolic standards (caffeic acid, chlorogenic acid, ferulic acid, gallic acid, p-coumaric acid, p-hydroxybenzoic acid, p-hydroxyphenylacetic acid, protocatechuic acid, sinapic acid, syringic acid, vanillic acid, catechin, eriodictyol, isorhamnetin 3-O-rutinoside, kaempferol 7-O-glucoside, naringenin, naringin, phloretin, phlorizin, quercetin, quercetin 3-O-glucoside, quercetin 3-O-rhamnoside, and rutin) were from Sigma-Aldrich (Steinheim, Germany). The fatty acid standard Supelco^®^ 37 Component FAME Mix was purchased from Sigma-Aldrich, Merck (Burlington, MA, USA). All reagents and chemicals whose purity was not previously stated were of analytical purity grade.

### 2.2. Mushroom Material

Pseudosclerotium of the mushroom *I. obliquus* was collected from birch trees (*Betula* spp.) in the fall of 2017, from the forest area of Mongolia (IM) and the Vlasina mountain, Republic of Serbia (IS). Considering the taxonomic characteristics and standard descriptions that can be found in monographs, the authors confirmed the belonging of the found pseudosclerotium to this species based on the examination of macro- and micromorphological features. For this research, pseudosclerotium was first dried to a constant mass in a stream of hot air (40 °C) and then ground to a fine powder by a Cyclotech mill (Tecator, Hoganas, Sweden) using a 0.5 mm sieve. The material was stored in the dark, in a cool and dry place until analysis. Representative specimens of *I. obliquus* pseudosclerotium were deposited in the collection of the Department of Industrial Microbiology, University of Belgrade—Faculty of Agriculture. Mycelium cultures of these fungi were also stored (at 4 °C) in the collection of the same Department.

### 2.3. Green Extraction Techniques

For the convenience of following the text more easily, the abbreviations for each set of origin of material, type of extraction, and extraction conditions are given in Table 1.

#### 2.3.1. Subcritical Water Extraction

Subcritical water extraction was performed in a batch-type high-pressure extractor (Parr 4520, Hillsboro, OR, USA), with a volume of 2 dm^3^. The reactor was equipped with an anchor stirrer and an electric heater that allowed the reaction mixture to be heated up to 350 °C. In all experimental runs, the finely divided material was mixed with water at a ratio of 1:10 (g/mL), and nitrogen was injected into the extractor to prevent possible oxidation at high temperatures in the presence of oxygen from the air. Extractions were conducted at temperatures of 120 °C and 200 °C, while the pressure (30 bar) was held constant during the 20 min extraction time. Extracts were filtered through filter paper under a vacuum, collected into glass flasks, and stored at 4 °C in a dark place until analysis.

#### 2.3.2. Ultrasound-Assisted Extraction

Ultrasound-assisted extraction was performed in a sonication water bath (EUP540A, Euinstruments, Paris, France). The bath consisted of a rectangular container with a frequency fixed at 40 kHz. Sonication was performed at a temperature of 30 °C, with 96% ethanol, 50% ethanol (*w*/*w*), and water and a solid/liquid ratio of 1:10 g/mL. Ultrasonic power (60 W/L) and extraction time (60 min) were kept constant. After the extraction, extracts were filtered through filter paper under vacuum, collected into glass vials, and stored in a dark place at 4 °C before analysis.

#### 2.3.3. Microwave-Assisted Extraction

Mono-mode microwave-assisted extraction was performed in a homemade setup consisting of a microwave oven (NN-E201W, Panasonic, Kadoma, Japan) and a suitable round-flask glass apparatus with a condenser. Extractions were performed with 96% ethanol, 50% ethanol (*w*/*w*), and water at an irradiation power of 470 W. The solid/liquid ratio (1:10, g/mL), extraction time (30 min), and frequency (50 Hz) were constant in all extractions. After extraction, the extracts were immediately filtered through filter paper under vacuum, collected into glass flasks, and stored at 4 °C until analysis.

### 2.4. Determination of the Total Polysaccharide Content

Total polysaccharide content (TPS) was measured by the phenol-sulfuric acid method with D-glucose as a reference [19]. The total polysaccharide content of extracts was expressed as glucose equivalents (GLUs) in g/100 g of dry weight of the extracts. A UV 1800 spectrophotometer (Shimadzu, Kyoto, 243 Japan) was used for spectrophotometric measurements.

### 2.5. Determination of Total Glucan, α- and β-Glucan Content

Total glucans (TGs) were assessed in the extracts using the Mushroom and Yeast β-glucan Assay K-YBGL09/2009 (Megazyme Int., Wicklow, Ireland). Contents of total glucans and α-glucans were calculated by comparing them with the D-glucose standard. The β-glucan content was calculated by subtracting the α-glucan from the total glucan content.

### 2.6. HPAEC/PAD Analysis of Soluble Free Sugars and Sugar Alcohols

The soluble sugar composition of different Chaga extracts was determined using HPAEC/PAD, according to the methodology previously described by Gašić et al. [20]. Briefly, a DIONEX ICS 3000 DP liquid chromatography system (Dionex, Sunnyvale, CA, USA) equipped with a quaternary gradient pump (Dionex), an ICS AS-DV 50 autosampler (Dionex), and a Carbo Pac^®^PA100 pellicular anion exchange column (4 × 250 mm, particle size 8.5 μm, pore size microporous, <10 A (Dionex)), was used for sugar analysis at 30 °C. The electrochemical detector consisted of gold as working and Ag/AgCl as reference electrodes. The mobile phase consisted of the following reagents: 600 mM sodium hydroxide (A), 500 mM sodium acetate (B), and ultrapure water (C). The linear gradient (flow rate, 0.7 mL/min) was as follows: 0–5 min, 15% A, 85% C; 5.0–5.1 min, 15% A, 2% B, 83% C; 5.1–12.0 min, 15% A, 2% B, 83% C; 12.0–12.1 min, 15% A, 4% B, 81% C; 12.1–20.0 min 15% A, 4% B, 81% C; 20.0–20.1 min 20% A; 20% B; 60% C; 20.1–30.0 min 20% A; 20% B; 60% C. Before analyses, the system was preconditioned with 15% A and 85% C for 15 min. The sample injection volume was 25 μL. Quantification of carbohydrate concentration was obtained from calibration curves of pure compounds, as already reported by Gašić et al. [20].

### 2.7. Total Phenolic Compounds Content

The Folin–Ciocalteu reaction method adapted for a 96-well microplate reader (microplate reader ELx808, BioTek Instruments, Inc., Winooski, VT, USA) was used to determine total phenol content (TPC) according to [21]. The results were expressed as gallic acid equivalents (GAEs) in g/100 g dry weight of the extracts. The absorbance was read at 630 nm.

### 2.8. Phenolic Profile

The phenolic compounds were each identified and quantified by UHPLC–DAD–MS/MS. Working solutions of phenolic standards (0.01; 0.05; 0.10; 0.25; 0.50; 0.75; 1.00 mg/L) were obtained by diluting the stock solution (1000 mg/L) prepared in methanol with a mobile phase (0.1% acetic acid (eluent A) in ultrapure water and acetonitrile (eluent B)) and stored in the dark at 4 °C. Separation and quantification of polyphenols was performed using a Dionex Ultimate 3000 UHPLC system equipped with a diode array detector connected to a TSQ Quantum Access Max triple-quadrupole mass spectrometer (Thermo Fisher Scientific, Basel, Switzerland) with an ion source in the form of electrospray ionization (200 °C) in the negative mode (from 100 to 1000 *m*/*z)*—triple quadrupole (UHPLC-DADMS/MS). The spraying voltage was 5 kV and the capillary temperature was 300 °C. The following conditions were previously described by Gašić et al. [22]. Chromatography was performed at 40 °C on a Syncronis C18 column (100 × 2.1 mm, 1.7 μm particle size) as follows: 0.0–1.0 min 5% B, 1.0–16.0 min from 5% to 95% (B), 16.0–16.1 min from 95% to 5% (B), then 5% (B) for 4 min. The flow rate was 0.300 mL/min and the DAD detector wavelengths were 254 and 280 nm. For quantification of polyphenols for each standard, the molecular ion and the two most intense fragments of the MS 2 spectra were recorded. Xcalibur software (version 2.2) was used to control the instrument. Phenolic compounds were identified by direct comparison with commercial standards. The total amount of each compound was calculated from the respective calibration curves and expressed as per mg/kg of dry weight of the extracts.

### 2.9. Determination of Protein Content

The Bradford method as described by Doroški et al. [22] was adapted for a 96-well microplate reader (ELx808 microplate reader, BioTek Instruments, Inc., USA). Bovine serum albumin (BSA) was used as a standard. The total extracted protein (TEP) in the extracts was expressed as g of BSA equivalents per 100 g of dry weight of the extracts.

### 2.10. Gas Chromatography–Mass Spectrometry

The fatty acid extract was dried and weighed. The content of unsaturated fatty acid was determined by the standard pharmacopeia method whereas the saturated content was determined as the difference between the total and unsaturated. GC/MS analysis of fatty acids was conducted using a Thermo Fisher’s (Waltham, MA, USA) Focus GC coupled to a Polaris Q ion trap MS detector. The analysis was performed using a well-known and described method [23]. Compounds were identified by comparing their mass spectra with those in the NIST database and with those obtained from standards. Final results were expressed as per g/100 g of dry weight of the extracts.

### 2.11. Statistical and Principal Component Analysis

SPSS Statistics 17.0 was used for statistical data analysis. The results were presented as means of three measurements ± standard deviation. Differences between mean values were estimated using Tukey’s post hoc tests and the least significant difference (LSD), at a significance level of *p* < 0.05.

Principal component analysis (PCA) was performed using RStudio v2022.07.2 on yield data and 51 chemical parameters (22 phenolic compounds with total phenolic content and 23 sugars and their total content) obtained with three types of extraction. The data were scaled and centered through the prcomp function from the stats R package and plotted with the ggbiplot library.

## 3. Results

### 3.1. Extraction Yield, Total Proteins, Total Polysaccharides, Total, α- and β-Glucans, and Total Phenolic Compounds

The average yield for each extract was calculated and presented in Table 2. The highest yield was obtained for the IM SWE 120 °C (33.7 ± 1.10 g/100 g), IM SWE 200 °C (21.27 ± 2.46 g/100 g), IS SWE 120 °C (20.52 ± 0.79 g/100 g), and SWE 200 °C (18.93 ± 0.23 g/100 g) samples. For both materials, SWE under lower temperature gave a significantly higher yield than any other technique. On the other hand, the extraction with 96% EtOH (both ultrasound and microwave-assisted) gave the lowest yield; IS MW (0.3 ± 0.10 g/100 g), IM VAE (0.9 ± 0.34 g/100 g), IS VAE (1.06 ± 0.26 g/100 g), and IM MW (2.17 ± 0.31 g/100 g). In most cases, there was no statistical difference between the yield of Chaga from Mongolia and Serbia when the same extraction technique was performed.

The TEP content ranged from 7.08 ± 0.13 to 26.82 ± 1.71 mg/g BSA. The samples with the highest protein content were obtained with water or 50% EtOH, while SWE, regardless of temperature, also gave high levels of total proteins. There was a significant difference in the TEP content when a different percentage of alcohol was used no matter if the method applied was ultrasound or microwaves. On the contrary, MW and VAE 96% EtOH resulted in the lowest concentration of proteins. The origin of the material had no influence.

The TPS content showed the highest level of variations depending on the extraction technique used (Table 2). The highest amount was determined in the SWE 200 °C IS (536.15 ± 39.54 mg/g GLU) and IM (580.28 ± 4.23 mg/g GLU) extracts. Microwave extraction combined with the higher concentration of alcohol gave the lowest yield of total polysaccharides in both kinds of Chaga (IS, 183.95 ± 17.28 mg/g GLU and IM, 146.90 ± 6.66 mg/g GLU). In general, water appeared as a better choice when considering the solvent regardless of the extraction technique. Like with the proteins and extract yield, whether the Chaga was from Mongolia or Serbia, the content of the extracted polysaccharides mostly did not differ significantly.

The results showed that the TG content in the Chaga extracts was strongly affected by the type of extraction and the choice of solvent. The highest percentage of TGs was obtained by SWE at 200 °C and was not statistically different for IS (20.43 ± 1.09 g/100 g) and IM (20.95 ± 0.83 g/100 g). Different strengths of ethanol strongly affected the extracted amount of glucans: 50% EtOH increased the TG concentration when compared with 96% EtOH. In this case, the origin of the material had an influence besides the choice of extraction technique and solvent. Individual components of interest, like α- and β-glucans, were present in relatively small amounts. SWE at 200 °C gave up to 20 times higher content of β-glucans than all the other techniques, but there was no difference between the IM and IS. A higher percentage of ethanol in combination with MW or VAE, for both the IM and IS, resulted in the lowest content of β-glucans (0.62 ± 0.01 and 1.13 ± 0.17 g/100 g, in the IS and IM, respectively). Similarly, SWE at 120 °C gave the highest percentage of α-glucans (IS 3.46 ± 0.05 and IM 2.50 ± 0.01 g/100 g) and significantly differed from the SWE at 200 °C. All the other techniques gave a similar amount of this polysaccharide.

In the case of total phenolics, combining 50% EtOH and microwaves resulted in the highest concentration (IS, 90.16 ± 1.51 mg/g GAE and IM, 55.65 ± 0.88 mg/g GAE). Evidently, the raw materials’ origin had a significant influence too. Also, a higher content of TPC was detected in the samples obtained by SWE, regardless of the extraction temperature. However, there was a significant difference between the TPC content extracted at 120 and 200 °C, in the IS and IM. A combination of water and ultrasound waves had the lowest extraction ability in the case of TPC (IS, 40.45 ± 3.58 mg/g GAE and IM, 19.47 ± 0.49 mg/g GAE). The origin of the material could not be excluded, Serbian Chaga being richer in total phenolics. The influence of the origin of the material was important and consistent no matter which technique was applied.

### 3.2. Qualitative and Quantitative Analysis of Sugars

**Figure 1:**
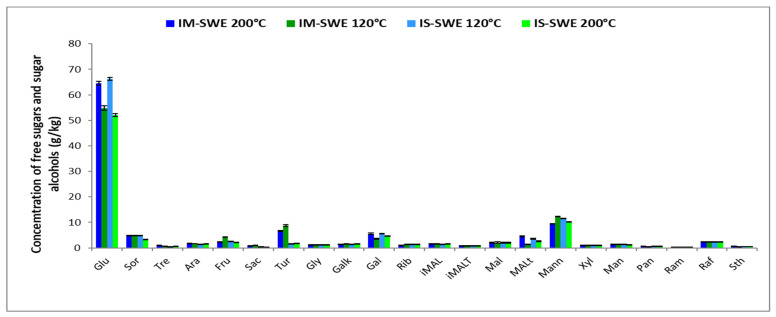
Content of different sugars detected in SWE-obtained extracts of *I. obliquus*; IM—Mongolian Chaga; IS—Serbian Chaga; SWE—subcritical water extraction at 120 and 200 °C; Sor—sorbitol; Tre—trehalose; Ara—arabinose; Glu—glucose; Fru—fructose; Sac—saccharose; Tur—turanose; Gly—glycerol; Galk—galactitol; Gal—galactose; Rib—ribose; iMAL—isomaltose; iMALt—isomaltotriose; Mal—maltose; MALt—maltotriose; Mann—mannitol; Xyl—xylose; Man—mannose; Pan—panose; Ram—ramnose; Raf—raffinose; Sth—stachyose.

The soluble sugar profile and their quantities in the SWE-obtained extracts of IS and IM are presented in Figure 1. as the most representative. Sugar profiles for other extraction techniques are given in the Supplementary Materials (Tables S1 and S2). According to the results of the HPAEC analysis, 22 different sugars were found in all the analyzed samples. Monosaccharides were present in significantly higher concentrations while only traces of di- and trisaccharides were detected. The presumed presence of monosaccharides, such as glucose, fructose, and galactose was confirmed. The highest amount of glucose was detected in the SWE samples, especially those extracted under 200 °C (64.545 ± 0.733 g/kg in the IM and 66.308 ± 0.593 g/kg in the IS). The origin of the samples had no influence, except in the case of turanose. Glucose was present ranging from 16.789 ± 0.316 to 28.632 ± 0.416 g/kg in the MW and VAE samples; water appeared as a better choice when considering the solvent in these samples. Other sugars that were extracted by the SWE technique in concentrations higher than a few g/kg, were sorbitol, turanose, galactose, maltotriose, and mannitol, as seen in Figure 1. When compared with all the techniques applied, SWE stood out as the most efficient technique for several individual sugars extraction. A higher concentration of fructose was present in the samples obtained by microwave and ultrasound-assisted extraction, especially when combined with water (IS VAE, 16.856 ± 0.176 and IM VAE, 12.536 ± 0.221 g/kg; IS MW, 14.653 ± 0.388 and IM MW, 11.356 ± 0.168 g/kg). In most of the cases, Serbian Chaga contained significantly higher amounts of fructose. A significant difference in sugar content between the Serbian and Mongolian Chaga was recorded in the case of galactose and saccharose. This was especially present when other techniques than SWE were used. At the same time, SWE extraction gave significantly lower amounts of saccharose in both Chaga extracts. However, Chaga from Mongolia contained high quantities of saccharose, ranging from 5 to more than 13 g/kg. For comparison, saccharose concentration in the extracts from Serbia did not reach even the lower level of the same sugar in the Mongolian samples. A higher concentration of sugar alcohol sorbitol was detected in the SWE extracts, reaching almost 5 g/kg of material, in both the IM and IS (Figure 1). Other extraction techniques gave similar concentrations of sorbitol, mostly around 1.5 to 2.5 g/kg. Another sugar alcohol, mannitol, was also present in high concentration in the SWE extracts, regardless of the materials’ origin and extraction temperature; its highest concentration was 12.455 ± 0.005 g/kg in the Chaga originating from Mongolia. Other extraction methods were not efficient in extracting mannitol from the IS; the highest concentration was slightly above 1 g/kg in a sample obtained with VAE in water. The same extraction technique and solvent gave the highest concentration of mannitol from the IM, and it was significantly higher than in the IS (IM, 10.233 ± 0.016 g/kg and IS, 1.042 ± 0.006 g/kg). Glycerol was most efficiently extracted by microwaves, while the choice of solvent was less significant in the IS than in the IM. In addition, significantly smaller amounts of trehalose, isomaltose, isomaltotriose, maltotriose, mannose, and panose were detected in amounts under 1 g/kg, or even less than 0.1 g/kg in all the extracts, no matter which extraction technique was used.

### 3.3. Phenolic Compounds Profile

**Figure 2:**
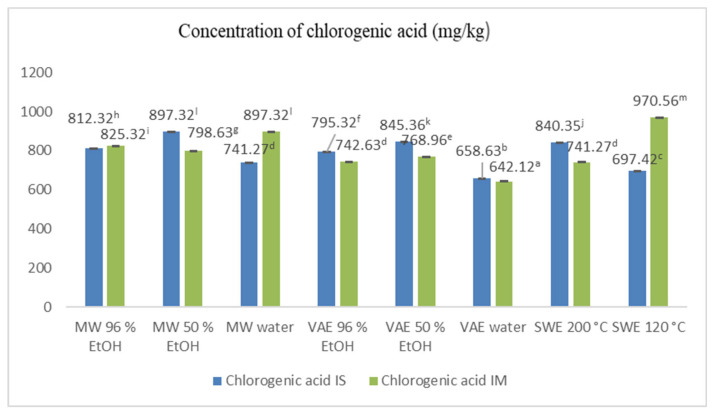
Chlorogenic acid content in *I. obliquus* extracts obtained by different green extraction techniques; IS—Serbian Chaga, IM—Mongolian Chaga, MW 96% EtOH, 50% EtOH, H_2_O—microwave-assisted extraction, VAE 96% EtOH, 50% EtOH, H_2_O—ultrasound-assisted extraction, SWE 200 °C, 120 °C—subcritical water extraction. Means in the bars with different lowercase letters are significantly different (*p* < 0.05).

Analysis of the phenolic composition of the Chaga extracts obtained by different green extraction techniques resulted in the identification and quantification of 23 different compounds which are presented in the Supplementary Materials (Tables S3 and S4). Chlorogenic acid stood out the most, being present in significantly higher concentrations than any other phenolic compound (Figure 2). It ranged from 741.27 to 970.56 mg/kg of extract, its content strongly dependent on the extraction technique, solvent, temperature, and place of the raw material’s origin. The highest concentration of chlorogenic acid was detected in the IM undergoing SWE at 120 °C while in the IS this was not the case (697.42 ± 0.93 mg/kg). Ultrasound-assisted extraction in water resulted in the lowest amount of this phenolic compound for both materials (IM, 642.12 ± 1.28 and IS, 658.63 ± 0.22 mg/kg of dry extract). Microwave extraction in ethanol of lower strength as well as ultrasound-assisted extraction with the same lower concentration of ethanol gave high amounts of chlorogenic acid, being significantly more efficient in the case of the Chaga from Serbia. The results of these two techniques were comparable and no significant difference was established.

Although both raw materials had the same qualitative profile, the concentrations of each phenolic compound varied significantly depending on the place of origin (Tables S3 and S4). Subcritical water extraction at 120 °C resulted in samples richer in protocatechinic, caffeic (IS), p-coumaric (IS), and cinnamic acid, while at 200 °C the same compounds were present in significantly lower concentrations. A flavonoid, catechin, was better extracted when microwave or ultrasound-assisted extraction was applied, in combination with ethanol (both tested concentrations were almost equally efficient), while the same methods in combination with water resulted in significantly less catechin. A significant difference was observed in the content of catechin depending on the origin of the material, the Mongolian Chaga extracts having more of it (almost double the amount), e.g., the IM MW with 95% EtOH had 5.99 ± 0.06 mg of catechin per g of dry extract while the IS that had undergone the same technique had 2.80 ± 0.02 mg/g. On the other hand, SWE was not the best choice for catechin, so the amounts recorded in this case were significantly lower (1.51 ± 0.05 mg of catechin per g of dry extract). Another flavonoid, quercetin, behaved similarly. Low amounts of it were extracted when subcritical water extraction was performed (in the case of both Chagas), while MW extraction using ethanol (both concentrations) resulted in significantly higher concentrations when compared with VAE. So, the order of efficacy was MW 50% EtOH > MW 96% EtOH > VAE 50% EtOH > VAE 96% EtOH. Water combined with MW or VA was in between VAE 50% EtOH and VAE 96% EtOH. Material from Mongolia contained more quercetin than the one from Serbia.

Caffeic and cinnamic acids were detected in larger amounts in the IM MW and VA-obtained extracts, with a slightly higher concentration when 50% EtOH was used. p-coumaric acid content was lower in the Mongolian than in the Serbian Chaga, regardless of the type of extraction. A low concentration of p-hydroxybenzoic and p-hydroxyphenylacetic acids was detected in all the extracts (IS and IM). Vanillic, syringic, sinapic, and ferulic acids were detected in moderate amounts, between 0.10 and 0.60 mg/kg, regardless of the extraction technique and origin of raw material. Gallic acid was found in the IM and IS extracts in the range of 1–2.69 mg/kg. SWE extraction resulted in significantly less content of gallic acid, 0.34–0.46 mg/kg, while the ultrasound-assisted extraction technique promoted its extraction the most, regardless of the material’s origin but dependent on the solvent and its concentration, in favor of EtOH of lower concentration.

The quantity of quercetin 3-O-glucoside, quercetin 3-O-rhamnoside, and kaempferol 7-O-glucoside was significantly (*p* < 0.05) higher in SWE (120 and 200 °C, IM and IS) when compared to the other treatments (MW and VAE) where they were found in traces. Flavonoids like phloretin and naringenin were detected in minimal amounts. Rutin and eridictyol showed a similar trend, although the concentrations were even lower.

### 3.4. Fatty Acid Profile

The results of the main fatty acids found in different types of extracts from *I. obliquus*, as well as the percentages of saturated fatty acids (SFAs) and unsaturated fatty acids (UFAs) are presented in Table 3. A total of 36 fatty acids were detected in all the samples (data shown in the Supplementary Materials, Tables S5 and S6). The UFAs were present at a much higher rate than the SFAs, especially when the extracts were obtained by microwave or ultrasound-assisted techniques combined with EtOH. Water, in general, resulted in half of the amount of UFAs obtained with EtOH. However, SWE at 200 °C produced samples whose ratio of UFA was comparable with other more efficient techniques. The concentration of EtOH had no significant influence on the UFAs. The fatty acids with the highest percentage in the IM and IS were palmitic (C16:0), stearic (C18:0), and oleic acid (C18:1 cis). The highest rate was observed for C18:1 cis, especially when the raw material underwent MW extraction, with comparable results for both concentrations of EtOH and the origin of the material. Water was not the adequate solvent since the percentage of C16:0, C18:0, and C18:1 cis was the lowest in all the tested samples and MW and VAE techniques. Both the IM and IS had similar quantities of the most abundant fatty acids.

Other fatty acids were represented with a share of less than 1 mg/100 g. As for the SFAs, palmitic acid was found in the highest amount (21.35 mg/100 g) in the Mongolian *I. obliquus* treated with SWE at 200 °C. The best option for extracting stearic acid was MW but also VAE with 96% or 50% EtOH, regardless of the materials’ origin. As for the UFAs, oleic acid was found in higher percentages in the Mongolian Chaga extracts obtained by MW 96% EtOH and 50% EtOH (52.32 and 55.63 mg/100 g, respectively), while in the Serbian Chaga, the extracts obtained with MW 96% EtOH and 50% EtOH resulted in 48.63 and 49.21 mg/100 g of oleic acid, respectively.

### 3.5. Principal Component Analysis (PCA)

**Figure 3:**
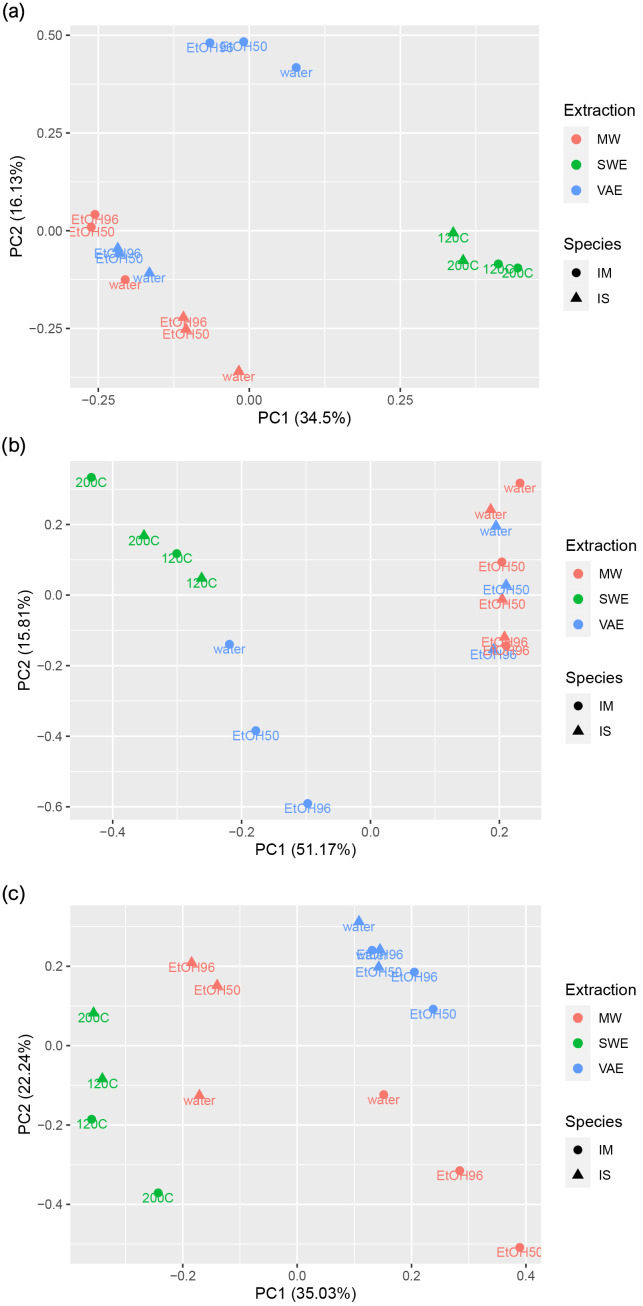
Principal component analysis for (**a**) the yield and total 51 examined compounds (**b**) TPC and phenolic compounds (**c**) TPS and sugar content; IS—Serbian Chaga, IM—Mongolian Chaga, MW 96% EtOH, 50% EtOH, H_2_O—microwave-assisted extraction, VAE 96% EtOH, 50% EtOH, H_2_O—ultrasound-assisted extraction, SWE 200 °C, 120 °C—subcritical water extraction. IM (point) and IS (triangle); TPCs, total phenolic compounds; TPSs, total polysaccharides; Different extraction techniques are expressed in different colors, while the names below point/triangle indicate the solvent/conditions used.

PCA based on the yield and a total of 51 analyzed compounds obtained through different extraction protocols from the Chaga, showed that the first principal component explained 34.50% of the data’s variation, with the second principal component contributing to a total explained variation of 50.63%. These data included yield, TPC, TPS, TG, α-glucans, β-glucans (Table 2); 23 sugars (Tables S1 and S2); 22 phenolic compounds (Tables S3 and S4), and three fatty acids (Table 3). The score plot (Figure 3a) shows the separation into three groups. The SWE samples of the IS and IM Chaga, regardless of temperature, clustered together and separated from the other samples. The second group consisted of the IM VAE-obtained extracts. The third group consisted of the other analyzed samples (IS VAE) and all MW (IS and IM). The overall results of the analysis of the composition of the Chaga extracts showed that the type of extraction expressed the highest degree of influence and not so much the choice of solvent and temperature. PCA based on data for TPS, TG, α-alfa glucans, β-beta glucans, and 23 sugars in the extracts obtained during three different extraction protocols, resulted in a two-component model that explained 66.98% of the total variance among the data shown in the score plot (Figure 3c). The samples were separated into three clusters on the score plot. The IS and IM SWE samples were separated together, while all the IM VAE-obtained samples, IS VAE, IS, and MW samples were clustered into two groups. PCA based on TPC and 22 phenolic compounds in different extracts of the Chaga resulted in a two-component model that explained 57.27% of the total variance among the data shown in the score plot (Figure 3b). Four clusters of samples could be observed in the score plot. The VAE samples (IS and IM Chaga) were separated from the other samples into one cluster. Also, the SWE samples accumulated in a special group. The MW samples were separated into two groups related to the material’s origin. The IS extracts formed one group, while the IM were in the second one in which differences were based on the origin of Chaga.

## 4. Discussion

In 2021, the prestigious Canadian Institute for Advanced Research organized a workshop attended by leading experts in fungal biology, which aimed to address the challenges and opportunities in the field of fungi having in mind both industry and academia [16]. Among the five avenues of future efforts in accelerating fungal research, novel medicines and enzymes derived from fungi were selected as an opportunity. Being exceptionally diverse, the fungal kingdom (especially Basidiomycota), is still under-studied and under-explored, with great prospects in the discovery of novel drugs and biologically active compounds with applications in medicine, food, feed, and dietary supplements. On the one hand, there is an increasing number of research papers screening different Basidiomycete species, in the context of biological activity in vitro, and a small but steady increase in the number of clinical studies with purified compounds such as mushroom-derived β-glucans [24]. The number of recorded medicinal effects reached more than 130 [25]. Mushrooms have been popularized in scientific and public circles, while laypeople are learning about the health benefits these organisms can provide when consumed as food and nutraceuticals. Demand is growing as the industry struggles to ensure quantity and quality. The road to an effective product that consumers can trust is slow and full of hardships. The industry is mainly left to navigate alone through the production, extraction, purification, and formulation of mushroom-based products. Moreover, the production cycle has to meet the SDGs and decrease the use of harmful chemicals and the CO_2_ footprint. This means paying more attention to alternatives and GETs. However, research in this field in combination with mushroom raw material is scarce. In this study, several GETs were examined as an option in line with the global requirements. To assess how different GETs affect chemical composition, the popular medicinal Chaga mushroom from Mongolia and Serbia was treated by microwave and ultrasound-assisted extraction with water or ethanol, and subcritical water extraction at different temperatures. Compounds of practical interest, which prove as valuable from the nutritive or medicinal aspects, were determined, and included total polysaccharides, proteins, phenolic compounds, glucans, α- and β-glucans, sugar profiles, and phenolic and fatty acid profiles, and their quantities. The relation between the yield of a specific compound and the extraction techniques was discussed with the final goal of making scientifically and industrially relevant recommendations.

As Simić et al. [26] reported, there can be significant differences in the extraction yield caused by different extraction techniques, the origin of raw material, climate conditions, as well as the choice of solvent. The highest yield was obtained when water was combined with high temperature and high pressure, as in the case of SWE. Wontcheu Fotso et al. [27] reported that Chaga extract yield was in the range of 0.02–0.03 g/g when they used ethanol while water significantly improved the yield (10 times) of the same material to 0.2–0.3 g/g. This suggests that more hydrophilic constituents might be present in Chaga mushroom. Several previously published reports have discussed different extraction yields of *I. obliquus*. Ma et al. [28] reported that the optimum SWE conditions to provide the highest yield (16.2 g/100 g) was 200 °C, an extraction time of 13 min, and a solid/liquid ratio of 1:30. A further increase in temperature (220 °C) resulted in a sharp decrease in the extraction yield. This was in accordance with our results; the optimal conditions for obtaining the highest yield, regardless of the origin of raw material, was 120 °C, with an extraction time of 20 min, and a solid/liquid ratio 1:10. As stated by Hu et al. [29], temperature is the most important factor in SWE. An elevated extraction temperature can modify the characteristics of the solvent and increase the rate of diffusion and solubility while decreasing viscosity and surface tension, which greatly contribute to the mass extraction of target compounds [30]. However, extremely high temperatures during the extraction process may cause structural degradation of various compounds. The solvent also plays an important role in SWE. Water, as a green solvent, is a highly polar and suitable molecule for industry due to its non-flammability, non-toxicity, low cost, and availability. Dielectric constant and polarity can be modified under critical conditions, enabling it to act as a solvent for non-polar compounds and behave similarly to organic solvents such as ethanol or methanol [13].

Proteins are important functional components in mushrooms, with increasing interest due to their pharmaceutical potential and use in developing functional foods [31]. In our study, the extracts with the highest protein content were obtained using water or 50% EtOH. The polar nature of water also makes it possible for use as an extraction solvent for water-soluble products such as proteins, sugars, and organic acids [13]. The lowest yield of TEP was determined in the samples obtained by MW or VAE techniques where a high concentration of ethanol was used. Similarly, Azad and Ping reported that water is a better option than alcohol when extracting proteins from mushrooms [32].

In an earlier study, Xi et al. [33] reported that increasing the temperature from 160 to 190 °C resulted in an increase in the content of polysaccharides from 85 mg/g to 132 mg/g. A further increase in temperature (up to 200 °C) resulted in the decrease in the polysaccharide content (100 mg/mL). In our study, as presented in Table 2, the TPS content also significantly increased with the increase in temperature from 120 to 200 °C. The same trend was observed in both kinds of material, the IM and the IS. In the study of Ma et al. [28], the yield of polysaccharides obtained from *I. obliquus* with the SWE technique was significantly higher (286.06%) in comparison with hot water extraction (3.66%). This was due to the lower solvent viscosity (water at elevated pressure and temperature above boiling point) and improved wetting ability providing effective mass transfer and a higher solubility of hydrophobic compounds [34]. On the other hand, the decrease in the polysaccharide content with increasing temperature can be attributed to the degradation and hydrolysis of these polymers when exposed to high temperatures during long extraction time, especially under subcritical water conditions. The resulting products cannot be detected in a phenol–sulphuric assay, thus, the final amount of polysaccharides is lower [35]. In addition to water, ethanol is often used in the extraction of polysaccharides to allow complete separation from other compounds such as lipids, phenols, and terpenes [17]. Other methods were not that efficient in polysaccharide extraction since the conditions applied were not enough to break the cell walls and hydrogen bonds in the raw material matrix.

Sugars are very important in cellular energy metabolism because they contribute to the proliferation of mushroom fruiting bodies [36]. The various beneficial properties of Chaga mushroom could be attributed to its sugar components. Hu et al. [29] showed that the biological activity of *I. obliquus* is mostly due to the presence of several polysaccharides, which consist of the following sugars: rhamnose, arabinose, xylose, mannose, glucose, and galactose. Additionally, it was reported that the polysaccharide extract of *I. obliquus* consists mainly of glucose (74.95%) and traces of rhamnose, arabinose, xylose, and galacturonic acid [37]. Xi et al. [33]. reported that the main components of the hot water Chaga extract were glucose and galactose, while the main components of the subcritical water Chaga extract were glucose, xylose, and mannose. This indicates that heat treatment under SWE conditions facilitates the degradation of galactose, but also increases xylose and mannose. On the other hand, Ma et al. [28] showed that the monosaccharide composition of SWE samples included xylose, fucose, and arabinose, with a small amount of glucose, mannose, galactose, and acid monosaccharide. This contrasts with our research where the highest amount of glucose was obtained in the subcritical water extracts. In earlier research, the hot water extract of Chaga was analyzed and different monomeric components such as xylose, rhamnose, mannose, glucose, inositol, and galactose were detected [36]. Also, Ma et al. [28] reported that in contrast to SWE polysaccharides, hot water extracts were mainly composed of rhamnose, fucose, arabinose, xylose, and galactose.

Dietary fructose intake has the potential to increase body weight and cause insulin resistance syndrome, hypertension, and hyperlipidemia in animal models [38]. In our study, the SWE samples had a low fructose content and, thus, are more suitable for dietetic supplements. The reasons for the differences in the qualitative analysis of sugars are not clear and may be related to different geographical environments, especially soil, host tree, and climate, which all affect the metabolism of the mushroom. Mingaila et al. [39] showed that the concentrations of Ca and Mg in the soil may have had a positive effect on the monosaccharide, as well as the total content of soluble solids in the studied birch sap. They reported that the sweetest and most nutritious sap is collected from birch stands growing in nutrient-rich soils that were temporarily flooded. Previously, Kuka et al. [40] reported that silver birch sap consists mainly of fructose (5.39 g/100 g), glucose (4.46 g/100 g), and sucrose (0.58 g/100 g). Thus, although not analyzed in this study, host trees from Serbia and Mongolia would probably differ in their chemical composition due to different environmental conditions. This will reflect on Chaga’s sugar composition as well, as already demonstrated in another study for Chaga originating from France, Canada, and Ukraine [41]. Strong evidence about the importance of the habitat on the birch sap composition, and not the species, was also reported by Grabek-Lejko et al. [42]. The authors stated that the total sugar content in birch sap increased from 1% in Finland to 2.6% in Poland.

Glucans, particularly β-glucan, are natural molecules with great therapeutic potential due to their immunomodulatory, antineoplastic, anti-inflammatory, antioxidant, anti-allergic, and antithrombotic activities [24]. However, they are most valued for their immunomodulatory activity [43]. Due to the stated reasons, glucans are of the greatest interest to the industry and can often be found on the label of mushroom-based nutraceuticals as a comparative advance. Moreover, cancer patients are prescribed very high doses, exceeding 2 g per day, of purified mushroom glucans. Thus, it is very important to develop the most efficient extraction technique for this compound. The majority of mushroom glucans are water soluble, and many techniques can facilitate and improve its extraction such as ultrasound-, microwave-, enzyme-assisted, and subcritical water extraction [44]. Our study showed that the content of total glucans in the Chaga extracts was significantly affected by the type of extraction, solvent choice, and temperature. The highest percentage of TGs was found when the material underwent SWE at 200 °C. At the same time, MW extraction resulted in several times a lower amount of β-glucans no matter which solvent was used. The same behavior was observed when the similarly hard structure of *Ganoderma lucidum* was treated with MW and pressurized liquid extraction [34]. In the study published by Jo et al. [45], the golden oyster mushroom SWE extract obtained at 200 °C for 60 min showed the highest content of β-glucan (12.84%). However, increasing the temperature of extraction from 250 to 300 °C caused its destruction. Yoo et al. [46] reported that a longer extraction time and higher temperature not only do not improve the extraction of β-glucan but also accelerate its decomposition. In another study, it was demonstrated that the extracts obtained by ultrasound had the lowest β-glucan content (3.02%) while the high temperature and pressure extract (5.88%), as well as the enzyme-obtained extract, contained higher amounts (5.86%) [47]. This agrees with our results.

Mushroom extracts are known to be rich in phenolics that contribute to various health benefits. Polyphenols are increasingly popular in the pharmaceutical industry and cosmetic formulations due to their widespread structural diversity and medicinal benefits [48]. Although the content of this group of molecules is still not mandatory on the product’s label, the industrial sector is aware of its significance, so the phenolics are part of the sought-after commercial preparations. Unlike other types of mushrooms, *I. obliquus* is attached to its host throughout its life and absorbs polyphenols from the tree it grows on [27]. The extraction of different phenolic compounds from the Chaga was highly dependent on the type of extraction, the solvent, and the raw materials’ origin. Combining 50% EtOH and microwaves produced the highest concentration of TPCs in our research. This is in agreement with previous reports indicating that the addition of water to organic solvents improves the relative polarity of the solvent allowing the solvents’ surface to interact with more phenolic compounds [49]. Boussetta et al. [50] reported that phenols show better solubility in the aqueous ethanol solution than in the anhydrous ethanol system. It has been demonstrated that exceeding the concentration of ethanol above 50% reduces the amount of polyphenols in the polysaccharide extract of *I. obliquus* [51]. Also, in this research, it was shown that SWE-treated material had a higher TPC. Extracts obtained at 200 °C contained a higher amount of TPC than those at 120 °C, which is a consequence of the increase in the dielectric constant of water. This means a higher solubility of non-polar compounds at higher temperatures, resulting in more phenolic compounds in the extract [52]. Other studies confirmed this: the extraction intensifies at higher temperatures reducing the solvent viscosity, allowing its better diffusion into the solid matrix, and increasing the solubility of phenolic compounds [53]. The same authors reported that the highest TPC content (10.72 mg/mL) was obtained in SWE extracts produced at 250 °C for 30 min, while it reached only 0.61 mg/mL when extracted at 50 °C for 10 min. Consistent with our results, SWE is an efficient technique to extract phenolic compounds from Chaga. In the context of obtaining the combined and popular full-spectrum supplements, coupling subcritical and ultrasonic extraction could provide a better yield of both groups of bioactive components [47].

In this study, it was observed that the IS compared to the IM for all the types of extractions yielded a higher amount of TPCs. However, the TPC content does not necessarily correlate with the potential biological effect of the extract, the profile and the amount of individual compounds being more important. Also, the Folin–Ciocalteu reagent, which has been used for years to determine total phenols, is considered non-specific because several non-phenolic substances such as sugars, ascorbic acids, and amino acids can interfere with TPC measurement [54]. Numerous reports of *I. obliquus* phenolic profile confirmed the presence of the following acids: protocatechuic, chlorogenic, caffeic, p-hydroxybenzoic, gallic, vanillic, syringic, and ferulic acids [55,56,57]. Chlorogenic acid is found in nature in many plants and fungi. This phenolic acid demonstrated several biological activities such as antioxidant, liver and kidney protection, anti-bacterial, anti-tumor, regulation of glucose and lipid metabolism, anti-inflammatory, and protection of the nervous system and blood vessels [58]. The amount of chlorogenic acid extracted by different extraction techniques was relatively similar due to its high solubility in ethanol and water [59]. However, pure water or ethanol was less efficient in extracting chlorogenic acid in general, which other authors have also reported [59].

The presence of this compound was significant in the Chaga from both countries. At the same time, chlorogenic acid as a component of this mushroom species has not been particularly emphasized in the existing literature. Here, we speculate that *I. obliquus* absorbed it from its host, the birch tree, which probably synthesized it in abundance as a response to harsh climate conditions and pathogen defense mechanisms. Chlorogenic acid is structurally highly diverse as a consequence of being an ester of caffeic and quinic acid [60]. It exists in the form of a monomer, dimer, or different glycosylated forms, as well as in different configurations [60]. It is not clear how different environmental conditions and the place of origin affect these forms. Still, their mere diversity leads to speculation that the Chaga from Serbia and Mongolia contained different metabolic forms of chlorogenic acid which affected its later extraction. As observed in the results section, MW and VAE 50% EtOH were the most efficient extraction techniques for the IS probably due to the presence of chlorogenic acid forms that easily dissolved in polar solvents like ethanol [58]. On the contrary, the IM might contain more glycosidic forms of chlorogenic acid decreasing its solubility in ethanol, thus, being readily extracted by SWE. The presence of caffeic, ferulic, and p-coumaric acids in significant amounts points to a possibility of the formation of mono-, di-, tri-, tetra-, and mixed esters which also belong to the group of chlorogenic acids. Together, they should positively impact the extract’s antioxidant and biological activity (a publication dedicated to this issue is under preparation). Thus, we suggest monitoring chlorogenic acids content in Chaga preparations since these compounds may exhibit health benefits such as in the case of diabetes, hypertension, and degenerative diseases [61].

Xi et al. [33] reported that SWE extraction of *I. obliquus* at 209 °C significantly increased the yield of phenolic compounds (477.58 mg GAL/g) compared to 75% ethanol extraction at 40 °C (121 mg GAL/g). In the same study, the amounts of phenolic compounds (expressed as mg/g dried material) in the extract of *I. obliquus* obtained with the SWE technique were as follows: 0.24 gallic acid, 8.57 epigallocatechin, 17.84 catechins, 1.99 chlorogenic acid, 1.07 vanillic acid, 13.33 epicatechin, and 1.60 ferulic acid, respectively.

Studies on SWE application on mushrooms so far have mostly focused on extracting phenolic compounds and polysaccharides [62]. However, previous research showed that SWE gives excellent results for the extraction of essential oils from herbs, indicating that the extraction of fatty acids from mushrooms by this GET is worthy of investigation [63]. Indeed, in our study, the 200 °C SW extracts contained a significant amount of unsaturated fatty acids especially when the extract was prepared from the IM.

Peng and Shahidi [64] identified 37 unmodified saturated/unsaturated fatty acids in Chaga samples. They confirmed the presence of lauric, myristic, palmoitoleic, linolenic, linoleic, stearic, palmitic, and oleic acids, similar to our research. On the other hand, Shcherbakov et al. [65] presented the qualitative and quantitative compositions (mg/100 g) of fatty acids in the same mushroom: lauric (0.2), miristic (0.2), palmitic (29.1), stearic (15.1), oleic (31.5), arachidic (4.6). They reported that palmitic, stearic, and oleic acids are present in the highest amounts, which aligns with our study. Currently, the industry mainly applies SWE to extract oil from the spores of *G. lucidum*. This oil is rich in unsaturated fatty acids; thus, it is recommended for patients with cardiovascular problems. Our results point out that the same technology can be applied in the case of Chaga, expanding the palette of products for the benefit of the consumers.

## 5. Conclusions

Being one of the most popular raw materials for mushroom supplements, Chaga is extensively processed and marketed mostly in the form of a dry extract (prepared by hot water extraction) or a double extract commercialized in the form of a tincture. These extraction techniques are neither green nor the most efficient when it comes to the yield of bioactive compounds and their composition. Thus, GETs can offer the best effectiveness–quality combination, for both scientists and industry. In this work, it has been demonstrated that subcritical water extraction, in the range of temperature from 120 to 200 °C, offers a win–win solution. SWE gave the highest yield of all the groups of important compounds, especially glucans and phenolics. Additionally, temperature, solvent, and the origin of the material appeared as important parameters since Chaga exploits the chemical makeup of its host tree. Current research on this mushroom’s medicinal benefits is expanding and being brought in connection with mushrooms’ chemical profile. Despite the extensive characterization of *I. obliquus* composition, the lipophilic fraction is still not well studied. As presented here, SWE is a good option for the extraction of this group of components as well, thus, extending the plethora of commercial products. Future Chaga preparations should focus on chlorogenic acids and unsaturated fatty acids, which together might have a synergistic effect when it comes to the issue of cardiovascular ailments.

## Figures and Tables

**Table 1:** Extract codes of Serbian and Mongolian *I. obliquus* extracts obtained by different green extraction techniques.

No.	Extraction Codes	Type of Extraction	Origin
1	IS MW 96% EtOH	microwave-assisted extraction	Chaga from Serbia
2	IS MW 50% EtOH	microwave-assisted extraction	
3	IS MW water	microwave-assisted extraction	
4	IS VAE 96% EtOH	ultrasound-assisted extraction	
5	IS VAE 50% EtOH	ultrasound-assisted extraction	
6	IS VAE water	ultrasound-assisted extraction	
7	IS SWE 200 °C	subcritical water extraction	
8	IS SWE 120 °C	subcritical water extraction	
9	IM MW 96% EtOH	microwave-assisted extraction	Chaga from Mongolia
10	IM MW 50% EtOH	microwave-assisted extraction	
11	IM MW water	microwave-assisted extraction	
12	IM VAE 96% EtOH	ultrasound-assisted extraction	
13	IM VAE 50% EtOH	ultrasound-assisted extraction	
14	IM VAE water	ultrasound-assisted extraction	
15	IM SWE 200 °C	subcritical water extraction	
16	IM SWE 120 °C	subcritical water extraction	

**Table 2:** Total yield and the amounts of total phenolic compounds, total extracted proteins, total polysaccharides, total glucans, and α- and β-glucan content of *I. obliquus* extracts.

Sample		Yield (g/100 g)	TEPs (mg/g BSA)	TPCs (mg/g GAE)	TPSs (mg/g GLU)	TGs (g/100 g)	α (g/100 g)	β (g/100 g)
IS	MW 96% EtOH	0.30 ± 0.10 ^a^	9.45 ± 2.32 ^ab^	72.79 ± 0.77 ^g^	183.95 ± 17.28 ^a^	4.85 ± 0.12 ^ef^	2.32 ± 0.12 ^g^	2.51 ± 0.01 ^cde^
	MW 50% EtOH	9.70 ± 0.09 ^de^	26.82 ± 1.47 ^f^	90.16 ± 1.51 ^h^	342.27 ± 49.86 ^cd^	6.71 ± 0.20 ^hi^	2.96 ± 0.08 ^i^	3.75 ± 0.12 ^fg^
	MW water	11.03 ± 0.63 ^ef^	19.42 ± 0.95 ^cd^	69.49 ± 0.75 ^g^	385.98 ± 3.96 ^d^	6.06 ± 0.01 ^gh^	2.83 ± 0.09 ^i^	3.24 ± 0.09 ^efg^
	VAE 96% EtOH	1.06 ± 0.26 ^ab^	7.56 ± 0.28 ^a^	63.21 ± 0.31 ^eg^	339.19 ± 30.89 ^cd^	2.65 ± 0.02 ^ab^	2.03 ± 0.01 ^ef^	0.62 ± 0.01 ^a^
	VAE 50% EtOH	3.60 ± 0.49 ^abc^	12.49 ± 0.37 ^b^	62.43 ± 0.60 ^eg^	297.58 ± 9.54 ^bc^	5.58 ± 0.10 ^fg^	2.55 ± 0.01 ^h^	3.03 ± 0.10 ^def^
	VAE water	4.45 ± 0.05 ^bc^	18.18 ± 0.66 ^cd^	40.45 ± 3.58 ^cd^	296.19 ± 6.76 ^bc^	6.11 ± 0.06 ^ghi^	1.83 ± 0.01 ^d^	4.28 ± 0.06 ^g^
	SWE 200 °C	18.93 ± 0.23 ^f^	17.95 ± 1.88 ^c^	89.94 ± 1.58 ^h^	536.15 ± 39.54 ^e^	20.43 ± 1.09 ^j^	1.43 ± 0.03 ^c^	19.00 ± 1.12 ^h^
	SWE 120 °C	20.52 ± 0.79 ^f^	20.68 ± 0.18 ^cde^	70.39 ± 15.09 ^g^	349.00 ± 23.84 ^cd^	7.20 ± 0.16 ^i^	3.46 ± 0.05 ^j^	3.74 ± 0.12 ^fg^
IM	MW 96% EtOH	2.17 ± 0.31 ^abc^	10.08 ± 0.22 ^ab^	32.56 ± 0.86 ^bc^	146.90 ± 6.66 ^a^	3.56 ± 0.18 ^bcd^	1.97 ± 0.05 ^de^	1.59 ± 0.13 ^abc^
	MW 50% EtOH	9.20 ± 4.0 ^de^	26.18 ± 1.71 ^f^	55.65 ± 0.88 ^ef^	400.87 ± 17.29 ^d^	3.80 ± 0.35 ^cde^	1.92 ± 0.07 ^de^	1.87 ± 0.28 ^bc^
	MW water	14.63 ± 2.75 ^f^	23.66 ± 0.11 ^ef^	47.81 ± 0.12 ^de^	306.71 ± 15.90 ^bc^	4.36 ± 0.13 ^de^	2.13 ± 0.04 ^f^	2.23 ± 0.17 ^cde^
	VAE 96% EtOH	0.90 ± 0.34 ^ab^	7.08 ± 0.13 ^a^	23.24 ± 3.38 ^ab^	249.90 ± 16.89 ^b^	2.17 ± 0.02 ^a^	1.05 ± 0.02 ^a^	1.13 ± 0.17 ^ab^
	VAE 50% EtOH	3.07 ± 0.34 ^abc^	21.10 ± 2.96 ^cde^	46.88 ± 0.90 ^de^	372.37 ± 1.29 ^d^	3.88 ± 0.09 ^cde^	1.27 ± 0.02 ^b^	2.61 ± 0.11 ^cde^
	VAE water	3.80 ± 0.10 ^abc^	19.76 ± 1.55 ^cde^	19.47 ± 0.49 ^a^	292.41 ± 11.92 ^bc^	3.20 ± 0.08 ^abc^	1.21 ± 0.01 ^b^	1.99 ± 0.08 ^bcd^
	SWE 200 °C	21.27 ± 2.46 ^f^	22.13 ± 1.09 ^de^	70.4 ± 0.97 ^g^	580.28 ± 4.23 ^f^	20.95 ± 0.83 ^j^	1.34 ± 0.02 ^bc^	19.61 ± 0.8 ^h^
	SWE 120 °C	33.70 ± 1.10 ^h^	20.53 ± 0.48 ^cde^	55.33 ± 0.74 ^ef^	354.81 ± 7.65 ^d^	4.15 ± 0.13 ^cde^	2.50 ± 0.01 ^gh^	1.67 ± 0.13 ^abc^

The results are expressed as the mean of three replicates ± standard deviation. Means in the same row with different lowercase letters differed significantly (*p* < 0.05). Abbreviations: IS—Serbian Chaga, IM—Mongolian Chaga, MW 96% EtOH, 50% EtOH, H_2_O—microwave-assisted extraction, VAE 96% EtOH, 50% EtOH, H_2_O—ultrasound-assisted extraction, SWE—subcritical water extraction. TEPs, total extracted proteins; TPCs, total phenolic compounds; TPSs, total polysaccharides; TGs—total glucans; α—alfa glucans, β—beta glucans.

**Table 3:** Fatty acid composition (mg/100 g) of *I. obliquus* extracts obtained by different green extraction techniques.

Sample		C16:0	C18:0	C18:1 cis	SFAs	UFAs
IS	MW 96% EtOH	18.32 ± 0.12 ^e^	6.58 ± 0.20 ^ef^	52.32 ± 0.58 ^h^	29.40 ± 0.19 ^f^	57.96 ± 0.58 ^g^
	MW 50% EtOH	16.22 ± 0.15 ^d^	6.96 ± 0.30 ^efg^	55.63 ± 0.79 ^i^	27.61 ± 0.30 ^e^	61.04 ± 0.79 ^h^
	MW water	15.66 ± 0.10 ^cd^	3.12 ± 0.10 ^b^	12.36 ± 0.13 ^a^	20.89 ± 0.10 ^c^	15.24 ± 0.13 ^a^
	VAE 96% EtOH	19.32 ± 0.32 ^e^	7.56 ± 0.26 ^hi^	38.25 ± 0.99 ^ef^	32.19 ± 0.32 ^h^	43.46 ± 0.99 ^e^
	VAE 50% EtOH	18.88 ± 0.41 ^e^	7.99 ± 0.38 ^i^	34.52 ± 1.50 ^d^	32.14 ± 0.41 ^h^	39.07 ± 1.49 ^d^
	VAE water	14.32 ± 0.23 ^b^	4.11 ± 0.21 ^c^	14.22 ± 0.32 ^a^	21.07 ± 0.23 ^c^	16.80 ± 0.32 ^a^
	SWE 200 °C	18.65 ± 0.28 ^e^	5.63 ± 0.11 ^d^	25.63 ± 0.43 ^c^	30.97 ± 0.28 ^g^	29.98 ± 0.43 ^c^
	SWE 120 °C	16.52 ± 0.19 ^d^	4.25 ± 0.10 ^c^	12.36 ± 0.12 ^a^	25.61 ± 0.19 ^d^	15.12 ± 0.12 ^a^
IM	MW 96% EtOH	14.63 ± 0.23 ^bc^	6.42 ± 0.23 ^e^	48.63 ± 1.41 ^g^	25.88 ± 0.23 ^d^	53.95 ± 1.41 ^f^
	MW 50% EtOH	13.89 ± 0.29 ^b^	6.56 ± 0.16 ^ef^	49.21 ± 1.64 ^g^	25.93 ± 0.29 ^d^	54.59 ± 1.64 ^f^
	MW water	10.32 ± 0.15 ^a^	3.88 ± 0.12 ^c^	21.33 ± 0.52 ^b^	16.48 ± 0.15 ^a^	24.99 ± 0.52 ^b^
	VAE 96% EtOH	15.63 ± 0.06 ^cd^	7.01 ± 0.13 ^fgh^	36.52 ± 1.68 ^de^	27.69 ± 0.13 ^e^	39.44 ± 1.68 ^d^
	VAE 50% EtOH	16.01 ± 0.05 ^d^	7.23 ± 0.16 ^gh^	39.78 ± 1.30 ^f^	28.20 ± 0.16 ^e^	42.76 ± 1.30 ^e^
	VAE water	10.63 ± 0.14 ^a^	4.11 ± 0.08 ^c^	14.33 ± 0.16 ^a^	17.64 ± 0.14 ^b^	16.09 ± 0.16 ^a^
	SWE 200 °C	21.35 ± 0.08 ^f^	2.53 ± 0.05 ^a^	35.85 ± 0.88 ^de^	30.77 ± 0.80 ^g^	40.65 ± 0.88 ^de^
	SWE 120 °C	18.63 ± 0.87 ^e^	2.11 ± 0.07 ^a^	12.23 ± 0.38 ^a^	25.58 ± 0.87 ^d^	15.45 ± 0.38 ^a^

The results are expressed as mg/100 g and the mean values of three replicates ± standard deviation. Means in the same row with different lowercase letters are significantly different (*p* < 0.05). The difference to 100% corresponds to other less abundant fatty acids (data shown in the Supplementary Materials, Tables S5 and S6). IS—Serbian Chaga, IM—Mongolian Chaga, MW 96% EtOH, 50% EtOH, H_2_O—microwave-assisted extraction, VAE 96% EtOH, 50% EtOH, H_2_O—ultrasound-assisted extraction, SWE 200 °C, 120 °C—subcritical water extraction. Palmitic acid (C16:0); Stearic acid (C18:0); Oleic acid (C18:1 cis); SFAs—saturated fatty acids; UFAs—unsaturated fatty acids.

## Data Availability

Data are contained within the article and Supplementary Materials. In addition, data is available upon request.

## Supplementary Materials

The following supporting information can be downloaded at: https://www.mdpi.com/article/10.3390/jof10030225/s1, Table S1: Free sugar and sugar alcohol profile of Mongolian *I. obliquus* extracts obtained by different green extraction techniques (g/kg); Table S2: Free sugar and sugar alcohol profile of Serbian *I. obliquus* extracts obtained by different green extraction techniques (g/kg); Table S3:. Polyphenol profile of Serbian *I. obliquus* extracts obtained by different green extraction techniques (mg/kg); Table S4: Polyphenol profile of Mongolian *I. obliquus* extracts obtained by different green extraction techniques (mg/kg); Table S5: Fatty acid profile of Serbian *I. obliquus* extracts obtained by different green extraction techniques mg/100 g; Table S6: Fatty acid profile of Mongolian *I. obliquus* extracts obtained by different green extraction techniques mg/100 g.

## References

[1] Arshadi N., Nouri H., Moghimi H. (2023). Increasing the Production of the Bioactive Compounds in Medicinal Mushrooms: An Omics Perspective. Microb. Cell Fact..

[2] https://ourworldindata.org/emissions-by-sector.

[3] https://www.epa.gov/trinationalanalysis/greenhouse-gas-reporting-chemical-manufacturing-sector.

[4] https://www.un.org/en/climatechange/what-is-climate-change.

[5] Bradu P., Biswas A., Nair C., Sreevalsakumar S., Patil M., Kannampuzha S., Mukherjee A.G., Wanjari U.R., Renu K., Vellingiri B. (2023). Recent Advances in Green Technology and Industrial Revolution 4.0 for a Sustainable Future.

[6] https://ec.europa.eu/research-and-innovation/en/projects/success-stories/all/green-manufacturing-pharmaceutical-industry.

[7] Hossain M.F. (2022). Green science and technology for designing sustainable world. Sustainable Design for Global Equilibrium.

[8] Vunduk J., Tura D., Biketova A.Y., Dhull S.B., Bains A., Chawla P., Sadh P.K. (2022). Chapter 4: Medicinal mushroom nutraceutical commercialization: Two sides of a coin. Wild Mushrooms Characteristics, Nutrition, and Processing.

[9] Tiwari B.K. (2015). Ultrasound: A clean, green extraction technology. TrAC Trends Anal. Chem..

[10] Mishra J., Khan W., Ahmad S., Misra K. (2021). Supercritical Carbon Dioxide Extracts of *Cordyceps Sinensis*: Chromatography-Based Metabolite Profiling and Protective Efficacy Against Hypobaric Hypoxia. Front. Pharmacol..

[11] Majid I., Khan S., Aladel A., Dar A.H., Adnan M., Khan M.I., Mahgoub Awadelkareem A., Ashraf S.A. (2023). Recent Insights into Green Extraction Techniques as Efficient Methods for the Extraction of Bioactive Components and Essential Oils from Foods. CYTA J. Food.

[12] Chemat F., Vian M.A., Cravotto G. (2012). Green Extraction of Natural Products: Concept and Principles. Int. J. Mol. Sci..

[13] Fraterrigo Garofalo S., Tommasi T., Fino D. (2021). A Short Review of Green Extraction Technologies for Rice Bran Oil. Biomass Convers. Biorefinery.

[14] Raj B., John S., Chandrakala V., Kumari G.H., Kumar S. (2023). Green extraction techniques for phytoconstituents from natural products. Medicinal Plants.

[15] https://www.azothbiotech.com/mushrooms.

[16] Calleja-Gómez M., Castagnini J.M., Carbó E., Ferrer E., Berrada H., Barba F.J. (2022). Evaluation of Pulsed Electric Field-Assisted Extraction on the Microstructure and Recovery of Nutrients and Bioactive Compounds from Mushroom (*Agaricus bisporus*). Separations.

[17] Rodrigues Barbosa J., dos Santos Freitas M.M., da Silva Martins L.H., de Carvalho R.N. (2020). Polysaccharides of *Mushroom pleurotus* spp.: New Extraction Techniques, Biological Activities and Development of New Technologies. Carbohydr. Polym..

[18] Rodríguez-Seoane P., Torres Perez M.D., Fernández de Ana C., Sinde-Stompel E., Domínguez H. (2022). Antiradical and Functional Properties of Subcritical Water Extracts from Edible Mushrooms and from Commercial Counterparts. Int. J. Food Sci. Technol..

[19] Du Bois M., Gilles K.A., Hamilton J.K., Rebers P.A., Smith F. (1956). Colorimetric method for determination of sugars and related substances. Anal. Chem..

[20] Gašić U.M., Natić M.M., Mišić D.M., Lušić D.V., Milojković-Opsenica D.M., Tešić Ž.L., Lušić D. (2015). Chemical Markers for the Authentication of Unifloral *Salvia officinalis* L. Honey. J. Food Compos. Anal..

[21] Kozarski M.S., Klaus A.S., Vunduk J., Nikšić M.P. (2020). The Influence of Mushroom Coriolus Versicolor and Hazelnuts Enrichment on Antioxidant Activities and Bioactive Content of Dark Chocolate. Food Feed Res..

[22] Doroški A., Klaus A., Kozarski M., Cvetković S., Nikolić B., Jakovljević D., Tomasevic I., Vunduk J., Lazić V., Djekic I. (2021). The Influence of Grape Pomace Substrate on Quality Characterization of Pleurotus Ostreatus—Total Quality Index Approach. J. Food Process. Preserv..

[23] Micić D., Ðurović S., Riabov P., Tomić A., Šovljanski O., Filip S., Tosti T., Dojčinović B., Božović R., Jovanović D. (2021). Rosemary Essential Oils as a Promising Source of Bioactive Compounds: Chemical Composition, Thermal Properties, Biological Activity, and Gastronomical Perspectives. Foods.

[24] Kozarski M., Klaus A., van Griensven L., Jakovljevic D., Todorovic N., Wan-Mohtar W.A.A.Q.I., Vunduk J. (2023). Mushroom β-Glucan and Polyphenol Formulations as Natural Immunity Boosters and Balancers: Nature of the Application. Food Sci. Hum. Wellness.

[25] Wasser S.P. (2014). Medicinal Mushroom Science: Current Perspectives, Advances, Evidences, and Challenges. Biomed. J..

[26] Simić S., Aćimović M., Vidović S., Banožić M., Vladić J. (2021). Viola odorata: Influence of supercritical fluid extraction on the efficiency of ultrasound- and microwave-assisted extraction of bioactive compounds. Croat. J. Food Sci. Technol..

[27] Wontcheu Fotso Y.A., Ghazi S., Belkaid A., Soucy J., Tremblay L., Lamarre S., Clarisse O., Touaibia M. (2023). Extraction, Chemical Composition, Antiradical Capacity, and Photoprotective Effect of *Inonotus obliquus* from Eastern Canada. Nutraceuticals.

[28] Ma Y., Chu Z., Nan W., Zheng X., Zhao Y., Bai Y., Ma X., Ma R., Jia Y., Lü X. (2023). Optimization of Polysaccharide Extraction from *Inonotus obliquus* by Subcritical Water and Characterization of its Physiochemical Properties and Bioactivity. Soc. Sci. Res. Netw..

[29] Hu Y., Teng C., Yu S., Wang X., Liang J., Bai X., Dong L., Song T., Yu M., Qu J. (2017). *Inonotus obliquus* Polysaccharide Regulates Gut Microbiota of Chronic Pancreatitis in Mice. AMB Express.

[30] Thirugnanasambandham K., Sivakumar V., Maran J.P. (2015). Microwave-Assisted Extraction of Polysaccharides from Mulberry Leaves. Int. J. Biol. Macromol..

[31] González A., Cruz M., Losoya C., Nobre C., Loredo A., Rodríguez R., Contreras J., Belmares R. (2020). Edible Mushrooms as a Novel Protein Source for Functional Foods. Food Funct..

[32] Al Azad S., Ai Ping V.C. (2021). Comparison of Protein and Amino Acids in the Extracts of Two Edible Mushroom, *Pleurotus sajor-caju* and *Schizophyllum commune*. Adv. Biosci. Biotechnol..

[33] Yuan X. (2017). Subcritical Water Extraction and Characterization of Polysaccharides and Phenolic Compounds from *Inonotus obliquus*. Ph.D. Thesis.

[34] Smiderle F.R., Morales D., Gil-Ramírez A., de Jesus L.I., Gilbert-López B., Iacomini M., Soler-Rivas C. (2017). Evaluation of Microwave-Assisted and Pressurized Liquid Extractions to Obtain β-D-Glucans from Mushrooms. Carbohydr. Polym..

[35] Yue F., Zhang J., Xu J., Niu T., Lü X., Liu M. (2022). Effects of Monosaccharide Composition on Quantitative Analysis of Total Sugar Content by Phenol-Sulfuric Acid Method. Front. Nutr..

[36] Eid J.I., Das B. (2020). Molecular Insights and Cell Cycle Assessment upon Exposure to Chaga (*Inonotus obliquus*) Mushroom Polysaccharides in Zebrafish (*Danio rerio*). Sci. Rep..

[37] Jiang S., Shi F., Lin H., Ying Y., Luo L., Huang D., Luo Z. (2020). *Inonotus obliquus* Polysaccharides Induces Apoptosis of Lung Cancer Cells and Alters Energy Metabolism via the LKB1/AMPK Axis. Int. J. Biol. Macromol..

[38] Vieira V., Barros L., Martins A., Ferreira I.C.F.R. (2016). Nutritional and Biochemical Profiling of *Leucopaxillus candidus* (Bres.) Singerwild Mushroom. Molecules.

[39] Mingaila J., Čiuldiene D., Viškelis P., Bartkevičius E., Vilimas V., Armolaitis K. (2020). The Quantity and Biochemical Composition of Sap Collected from Silver Birch (Betula Pendula Roth) Trees Growing in Different Soils. Forests.

[40] Kuka M., Čakste I., Geršebeka E. (2013). Determination of Bioactive Compounds and Mineral Substances in Latvian Birch and Maple Saps. Proc. Latv. Acad. Sci. Sect. B Nat. Exact Appl. Sci..

[41] Géry A., Dubreule C., André V., Rioult J.P., Bouchart V., Heutte N., Eldin de Pécoulas P., Krivomaz T., Garon D. (2018). Chaga (*Inonotus obliquus*), a Future Potential Medicinal Fungus in Oncology? A Chemical Study and a Comparison of the Cytotoxicity Against Human Lung Adenocarcinoma Cells (A549) and Human Bronchial Epithelial Cells (BEAS-2B). Integr. Cancer Ther..

[42] Grabek-Lejko D., Kasprzyk I., Zaguła G., Puchalski C. (2017). The Bioactive and Mineral Compounds in Birch Sap Collected in Different Types of Habitats. Balt. For..

[43] Mirończuk-Chodakowska I., Kujawowicz K., Witkowska A.M. (2021). Beta-Glucans from Fungi: Biological and Health-Promoting Potential in the COVID-19 Pandemic Era. Nutrients.

[44] Sun Y., He H., Wang Q., Yang X., Jiang S., Wang D. (2022). A Review of Development and Utilization for Edible Fungal Polysaccharides: Extraction, Chemical Characteristics, and Bioactivities. Polymers.

[45] Jo E.K., Heo D.J., Kim J.H., Lee Y.H., Ju Y.C., Lee S.C. (2013). The Effects of Subcritical Water Treatment on Antioxidant Activity of Golden Oyster Mushroom. Food Bioprocess Technol..

[46] Yoo H.U., Ko M.J., Chung M.S. (2020). Hydrolysis of Beta-Glucan in Oat Flour during Subcritical-Water Extraction. Food Chem..

[47] Hwang A.Y., Yang S.C., Kim J., Lim T., Cho H., Hwang K.T. (2019). Effects of Non-Traditional Extraction Methods on Extracting Bioactive Compounds from Chaga Mushroom (*Inonotus obliquus*) Compared with Hot Water Extraction. Lwt.

[48] Janjušević L., Karaman M., Šibul F., Tommonaro G., Iodice C., Jakovljević D., Pejin B. (2017). The Lignicolous Fungus *Trametes versicolor* (L.) Lloyd (1920): A Promising Natural Source of Antiradical and AChE Inhibitory Agents. J. Enzyme Inhib. Med. Chem..

[49] Luthria D.L. (2012). Optimization of Extraction of Phenolic Acids from a Vegetable Waste Product Using a Pressurized Liquid Extractor. J. Funct. Foods.

[50] Boussetta N., Vorobiev E., Le L.H., Cordin-Falcimaigne A., Lanoisellé J.L. (2012). Application of Electrical Treatments in Alcoholic Solvent for Polyphenols Extraction from Grape Seeds. Lwt.

[51] Wold C.W., Gerwick W.H., Wangensteen H., Inngjerdingen K.T. (2020). Bioactive Triterpenoids and Water-Soluble Melanin from *Inonotus obliquus* (Chaga) with Immunomodulatory Activity. J. Funct. Foods.

[52] Ko M.J., Nam H.H., Chung M.S. (2020). Subcritical Water Extraction of Bioactive Compounds from Orostachys Japonicus A. Berger (Crassulaceae). Sci. Rep..

[53] Seo H.K., Lee S.C. (2010). Antioxidant Activity of Subcritical Water Extracts from Chaga Mushroom (*Inonotus obliquus*). Sep. Sci. Technol..

[54] Medina M.B. (2011). Determination of the Total Phenolics in Juices and Superfruits by a Novel Chemical Method. J. Funct. Foods.

[55] Abu-Reidah I.M., Critch A.L., Manful C.F., Rajakaruna A., Vidal N.P., Pham T.H., Cheema M., Thomas R. (2021). Effects of Ph and Temperature on Water under Pressurized Conditions in the Extraction of Nutraceuticals from Chaga (*Inonotus obliquus*) Mushroom. Antioxidants.

[56] Szymański M., Smolibowska J., Szymański A. (2019). An Investigation into the Relationships between Antioxidant Activity and Chemical Elements as Well as Polyphenolics in Fungal Fruiting Bodies Growing on Betula L. J. Elem..

[57] Glamočlija J., Ćirić A., Nikolić M., Fernandes Â., Barros L., Calhelha R.C., Ferreira I.C.F.R., Soković M., Van Griensven L.J.L.D. (2015). Chemical Characterization and Biological Activity of Chaga (*Inonotus obliquus*), a Medicinal “Mushroom”. J. Ethnopharmacol..

[58] Pan X., Jiang L., Chu Y., Gao S., Jiang X., Zhang Y., Chen Y., Luo S., Peng C. (2022). The Biological Activity Mechanism of Chlorogenic Acid and Its Applications in Food Industry: A Review. Front. Nutr..

[59] Oteef M.D.Y. (2022). Comparison of Different Extraction Techniques and Conditions for Optimizing an HPLC-DAD Method for the Routine Determination of the Content of Chlorogenic Acids in Green Coffee Beans. Separations.

[60] Ncube E.N., Mhlongo M.I., Piater L.A., Steenkamp P.A., Dubery I.A., Madala N.E. (2014). Analyses of Chlorogenic Acids and Related Cinnamic Acid Derivatives from Nicotiana Tabacum Tissues with the Aid of UPLC-QTOF-MS/MS Based on the in-Source Collision-Induced Dissociation Method. Chem. Cent. J..

[61] Silva N., Mazzafera P., Cesarino I. (2019). Should I Stay or Should I Go: Are Chlorogenic Acids Mobilized towards Lignin Biosynthesis?. Phytochemistry.

[62] Roselló-Soto E., Parniakov O., Deng Q., Patras A., Koubaa M., Grimi N., Boussetta N., Tiwari B.K., Vorobiev E., Lebovka N. (2016). Application of Non-Conventional Extraction Methods: Toward a Sustainable and Green Production of Valuable Compounds from Mushrooms. Food Eng. Rev..

[63] Zakaria S.M., Kamal S.M.M. (2016). Subcritical Water Extraction of Bioactive Compounds from Plants and Algae: Applications in Pharmaceutical and Food Ingredients. Food Eng. Rev..

[64] Peng H., Shahidi F. (2022). Qualitative Analysis of Secondary Metabolites of Chaga Mushroom (*Inonotus obliquus*): Phenolics, Fatty Acids, and Terpenoids. J. Food Bioact..

[65] Shcherbakov D.N., Kukina T.P., Elshin I.A., Panteleeva N.V., Teplyakova T.V., Salnikova O.I. (2022). GC-MS Analysis of Lipophilic Chaga Mushroom Constituents. AIP Conf. Proc..

