# Supplementary material for: The Effect of Green Extraction Technologies on the Chemical Composition of Medicinal Chaga Mushroom Extracts
Source: J Fungi (Basel). 2024 Mar 19;10(3):225. doi: 10.3390/jof10030225 (PMC10971126; doi:10.3390/jof10030225)
Supplement: Supplementary file 1 [file jof-10-00225-s001.zip › jof-2911906-supplementary.pdf]

**Table S1.** Free Sugar and sugar alcohol profile of Mongolian *I. obliquus* extracts obtained by different green extraction techniques (g/kg).

| g/kg | | IM-MW 96% ETOH | IM-MW 50% ETOH | IM-MW water | IM-VAE 96% ETOH | IM-VAE 50% ETOH | IM-VAE water | IM-SWE 200°C | IM-SWE 120°C |
|---|---|---|---|---|---|---|---|---|---|
| Sor | avg | 1.552 | 1.986 | 2.014 | 2.024 | 2.236 | 2.526 | 4.775 | 4.769 |
| | sd | 0.021$^{bc}$ | 0.034$^{e}$ | 0.022$^{e}$ | 0.053$^{e}$ | 0.085$^{f}$ | 0.102$^{g}$ | 0.039$^{i}$ | 0.118$^{i}$ |
| | RSD | 1.350 | 1.712 | 1.112 | 2.622 | 3.790 | 4.028 | 0.824 | 2.472 |
| Tre | avg | 0.256 | 0.335 | 0.825 | 0.785 | 0.896 | 0.994 | 1.107 | 0.606 |
| | sd | 0.003$^{ab}$ | 0.005$^{cd}$ | 0.009$^{i}$ | 0.013$^{i}$ | 0.020$^{j}$ | 0.022$^{k}$ | 0.031$^{l}$ | 0.004$^{g}$ |
| | RSD | 1.108 | 1.464 | 1.102 | 1.684 | 2.270 | 2.164 | 2.770 | 0.670 |
| Ara | avg | 0.452 | 0.501 | 0.689 | 1.087 | 1.552 | 1.583 | 1.715 | 1.645 |
| | sd | 0.010$^{d}$ | 0.011$^{d}$ | 0.017$^{e}$ | 0.023$^{f}$ | 0.032$^{h}$ | 0.026$^{h}$ | 0.042$^{j}$ | 0.033$^{ij}$ |
| | RSD | 2.170 | 2.104 | 2.462 | 2.136 | 2.046 | 1.316 | 2.470 | 1.992 |
| Glu | avg | 16.789 | 18.663 | 21.023 | 22.657 | 25.852 | 28.114 | 64.545 | 54.842 |
| | sd | 0.316$^{a}$ | 0.371$^{b}$ | 0.368$^{c}$ | 0.044$^{cd}$ | 0.036$^{f}$ | 0.571$^{h}$ | 0.733$^{k}$ | 0.924$^{j}$ |
| | RSD | 1.884 | 1.986 | 1.750 | 0.104 | 0.064 | 0.904 | 1.136 | 1.684 |
| Fru | avg | 7.996 | 8.322 | 11.356 | 4.332 | 6.332 | 12.536 | 2.262 | 4.277 |
| | sd | 0.079$^{d}$ | 0.110$^{d}$ | 0.168$^{f}$ | 0.062$^{b}$ | 0.104$^{c}$ | 0.221$^{g}$ | 0.062$^{a}$ | 0.107$^{b}$ |
| | RSD | 0.986 | 1.316 | 1.482 | 1.424 | 1.644 | 1.764 | 2.730 | 2.512 |
| Sac | avg | 5.663 | 8.652 | 13.223 | 5.214 | 7.896 | 10.336 | 0.931 | 0.990 |
| | sd | 0.151$^{f}$ | 0.210$^{h}$ | 0.415$^{j}$ | 0.128$^{f}$ | 0.204$^{g}$ | 0.312$^{i}$ | 0.027$^{b}$ | 0.025$^{b}$ |
| | RSD | 2.672 | 2.428 | 3.136 | 2.446 | 2.589 | 3.023 | 2.874 | 2.552 |
| Tur | avg | 1.332 | 1.542 | 1.986 | 0.908 | 1.425 | 1.789 | 6.623 | 8.758 |
| | sd | 0.042$^{bc}$ | 0.055$^{cd}$ | 0.061$^{e}$ | 0.037$^{a}$ | 0.063$^{bc}$ | 0.092$^{de}$ | 0.109$^{g}$ | 0.376$^{h}$ |
| | RSD | 3.185 | 3.582 | 3.050 | 4.117 | 4.413 | 5.123 | 1.648 | 4.290 |
| Gly | avg | 2.145 | 3.562 | 5.333 | 0.881 | 1.224 | 1.456 | 1.211 | 1.192 |
| | sd | 0.054$^{de}$ | 0.118$^{g}$ | 0.122$^{h}$ | 0.073$^{a}$ | 0.144$^{b}$ | 0.132$^{bc}$ | 0.035$^{b}$ | 0.117$^{ab}$ |
| | RSD | 2.523 | 3.321 | 2.285 | 8.285 | 11.784 | 9.095 | 2.900 | 9.785 |
| Galk | avg | 1.985 | 2.356 | 3.014 | 1.012 | 1.109 | 1.552 | 1.389 | 1.483 |
| | sd | 0.062$^{ef}$ | 0.055$^{g}$ | 0.091$^{h}$ | 0.024$^{a}$ | 0.023$^{a}$ | 0.052$^{bc}$ | 0.056$^{b}$ | 0.079$^{bc}$ |
| | RSD | 3.149 | 2.328 | 3.018 | 2.378 | 2.048 | 3.368 | 4.021 | 5.300 |
| Gal | avg | 2.992 | 3.145 | 4.025 | 0.986 | 1.537 | 3.665 | 5.545 | 3.572 |
| | sd | 0.159$^{d}$ | 0.251$^{de}$ | 0.179$^{fg}$ | 0.044$^{a}$ | 0.061$^{b}$ | 0.062$^{ef}$ | 0.240$^{l}$ | 0.151$^{ef}$ |
| | RSD | 5.300 | 7.984 | 4.456 | 4.456 | 3.984 | 1.692 | 4.332 | 4.228 |
| Rib | avg | 1.856 | 2.563 | 3.222 | 0.117 | 0.853 | 1.114 | 0.965 | 1.314 |
| | sd | 0.058$^{g}$ | 0.101$^{h}$ | 0.131$^{l}$ | 0.006$^{a}$ | 0.051$^{b}$ | 0.048$^{cd}$ | 0.038$^{bc}$ | 0.064$^{def}$ |
| | RSD | 3.140 | 3.944 | 4.056 | 5.370 | 6.028 | 4.272 | 3.970 | 4.884 |
| iMAL | avg | 0.837 | 0.968 | 1.025 | 0.223 | 0.573 | 1.063 | 1.588 | 1.616 |
| | sd | 0.058$^{e}$ | 0.058$^{f}$ | 0.073$^{f}$ | 0.012$^{b}$ | 0.020$^{cd}$ | 0.061$^{f}$ | 0.019$^{i}$ | 0.053$^{i}$ |
| | RSD | 6.970 | 5.992 | 7.136 | 5.504 | 3.552 | 3.874 | 1.224 | 3.256 |
| iMALT | avg | 0.585 | 0.677 | 0.985 | 0.658 | 0.985 | 1.112 | 0.791 | 0.809 |
| | sd | 0.027$^{bc}$ | 0.052$^{d}$ | 0.042$^{g}$ | 0.027$^{cd}$ | 0.046$^{g}$ | 0.059$^{h}$ | 0.004$^{f}$ | 0.031$^{f}$ |
| | RSD | 4.622 | 7.712 | 4.256 | 4.038 | 4.664 | 5.308 | 0.558 | 3.864 |
| Mal | avg | 0.351 | 0.552 | 0.789 | 1.221 | 1.527 | 2.356 | 2.022 | 2.054 |

| | | | | | | | | | |
|---|---|---|---|---|---|---|---|---|---|
| | sd | 0.003$^{a}$ | 0.007$^{abc}$ | 0.009$^{bc}$ | 0.059$^{de}$ | 0.095$^{e}$ | 0.141$^{f}$ | 0.185$^{f}$ | 0.287$^{f}$ |
| | RSD | 0.942 | 1.242 | 1.196 | 4.872 | 6.228 | 5.970 | 9.172 | 13.996 |
| MALt | avg | 0.008 | 0.009 | 0.012 | 1.114 | 1.983 | 2.999 | 4.564 | 1.355 |
| | sd | 0.000$^{a}$ | 0.000$^{a}$ | 0.000$^{a}$ | 0.028$^{b}$ | 0.007$^{c}$ | 0.005$^{e}$ | 0.196$^{g}$ | 0.024$^{b}$ |
| | RSD | 3.570 | 2.370 | 3.264 | 2.506 | 0.354 | 0.178 | 4.284 | 1.766 |
| Mant | avg | 1.631 | 1.896 | 2.014 | 5.632 | 7.662 | 10.233 | 9.525 | 12.455 |
| | sd | 0.036$^{c}$ | 0.059$^{d}$ | 0.043$^{d}$ | 0.125$^{e}$ | 0.153$^{f}$ | 0.016$^{h}$ | 0.003$^{g}$ | 0.005$^{j}$ |
| | RSD | 2.226 | 3.112 | 2.124 | 2.226 | 1.992 | 0.156 | 0.032 | 0.044 |
| Xyl | avg | 0.856 | 0.993 | 1.258 | 0.568 | 0.754 | 0.963 | 0.973 | 0.981 |
| | sd | 0.000$^{c}$ | 0.011$^{ef}$ | 0.015$^{h}$ | 0.008$^{a}$ | 0.015$^{b}$ | 0.019$^{ef}$ | 0.038$^{ef}$ | 0.037$^{ef}$ |
| | RSD | 0.018 | 1.136 | 1.228 | 1.482 | 1.986 | 1.986 | 3.926 | 3.748 |
| Man | avg | 0.072 | 0.088 | 0.102 | 0.885 | 0.993 | 1.563 | 1.327 | 1.339 |
| | sd | 0.004$^{a}$ | 0.004$^{a}$ | 0.006$^{a}$ | 0.036$^{b}$ | 0.056$^{c}$ | 0.059$^{e}$ | 0.031$^{d}$ | 0.029$^{d}$ |
| | RSD | 5.178 | 4.226 | 5.570 | 4.024 | 5.652 | 3.774 | 2.332 | 2.166 |
| Pan | avg | 0.011 | 0.018 | 0.021 | 0.425 | 0.558 | 0.658 | 0.625 | 0.542 |
| | sd | 0.000$^{a}$ | 0.001$^{a}$ | 0.001$^{a}$ | 0.011$^{b}$ | 0.016$^{cd}$ | 0.020$^{f}$ | 0.024$^{ef}$ | 0.022$^{c}$ |
| | RSD | 2.663 | 3.826 | 2.562 | 2.511 | 2.845 | 2.998 | 3.829 | 4.056 |
| Ram | avg | 1.109 | 1.526 | 1.996 | 0.223 | 0.356 | 0.452 | 0.388 | 0.345 |
| | sd | 0.017$^{f}$ | 0.032$^{i}$ | 0.030$^{j}$ | 0.003$^{a}$ | 0.005$^{b}$ | 0.006$^{c}$ | 0.001$^{bc}$ | 0.002$^{b}$ |
| | RSD | 1.559 | 2.119 | 1.527 | 1.183 | 1.376 | 1.282 | 0.298 | 0.721 |
| Raf | avg | 0.856 | 1.243 | 2.012 | 2.141 | 2.853 | 3.114 | 2.415 | 2.443 |
| | sd | 0.031$^{a}$ | 0.025$^{c}$ | 0.050$^{e}$ | 0.043$^{f}$ | 0.043$^{h}$ | 0.018$^{i}$ | 0.050$^{g}$ | 0.053$^{g}$ |
| | RSD | 3.663 | 2.012 | 2.498 | 2.002 | 1.496 | 0.578 | 2.056 | 2.166 |
| Sth | avg | 0.617 | 0.882 | 1.022 | 1.025 | 0.995 | 0.896 | 0.569 | 0.542 |
| | sd | 0.013$^{d}$ | 0.025$^{f}$ | 0.027$^{g}$ | 0.008$^{g}$ | 0.007$^{g}$ | 0.001$^{f}$ | 0.015$^{c}$ | 0.020$^{c}$ |
| | RSD | 2.163 | 2.845 | 2.611 | 0.762 | 0.661 | 0.057 | 2.668 | 3.663 |

Legend: Mean of three replicates ± standard deviation. Different letters in the same row indicate statistically significant difference at $p < 0.05$. RSD (relative standard deviation). Abbreviations: IM - Mongolian Chaga, MW 96% EtOH, 50% EtOH, $H_2O$ - microwave-assisted extraction, VAE 96% EtOH, 50% EtOH, $H_2O$ - ultrasound-assisted extraction, SWE 200 °C, 120 °C – subcritical water extraction; Sor - sorbitol; Tre - trehalose; Ara - arabinose ; Glu - glucose; Fru - fructose; Sac - saccharose; Tur - turanose; Gly - glycerol; Galk - galactitol; Gal - galactose; Rib - ribose; iMAL - isomaltose; iMALt isomaltotriosea; Mal - maltose; MALt - maltotriose; Mant - mannitol; Xyl - xylose; Man-mannose; Pan - panose; Ram - ramnose; Raf -raffinose; Sth - stachyose.

**Table S2.** Free Sugar and sugar alcohol profile of Serbian *I. obliquus* extracts obtained by different green extraction techniques (g/kg).

| **g/kg** | | **IS-MW 96% ETOH** | **IS-MW 50% ETOH** | **IS-MW water** | **IS-VAE 96% ETOH** | **IS-VAE 50% ETOH** | **IS-VAE water** | **IS-SWE 200°C** | **IS-SWE 120°C** |
|---|---|---|---|---|---|---|---|---|---|
| Sor | avg | 1.213 | 1.425 | 1.632 | 1.698 | 1.783 | 2.012 | 4.897 | 3.304 |
| | sd | 0.014$^{a}$ | 0.039$^{b}$ | 0.037$^{cd}$ | 0.067$^{cd}$ | 0.081$^{d}$ | 0.086$^{e}$ | 0.048$^{i}$ | 0.043$^{h}$ |
| | RSD | 1.164 | 2.704 | 2.294 | 3.970 | 4.536 | 4.298 | 0.972 | 1.304 |
| Tre | avg | 0.234 | 0.266 | 0.287 | 0.352 | 0.405 | 0.425 | 0.521 | 0.676 |
| | sd | 0.005$^{a}$ | 0.007$^{ab}$ | 0.006$^{bc}$ | 0.011$^{d}$ | 0.017$^{e}$ | 0.021$^{e}$ | 0.015$^{f}$ | 0.027$^{h}$ |
| | RSD | 1.950 | 2.648 | 1.926 | 3.136 | 4.164 | 4.824 | 2.850 | 3.926 |
| Ara | avg | 0.189 | 0.211 | 0.223 | 0.232 | 0.152 | 0.352 | 1.445 | 1.597 |
| | sd | 0.003$^{ab}$ | 0.001$^{ab}$ | 0.001$^{ab}$ | 0.005$^{b}$ | 0.003$^{a}$ | 0.008$^{c}$ | 0.038$^{g}$ | 0.053$^{hi}$ |
| | RSD | 1.336 | 0.410 | 0.370 | 1.976 | 1.706 | 2.198 | 2.642 | 3.304 |
| Glu | avg | 24.356 | 26.985 | 28.632 | 18.962 | 22.245 | 26.358 | 66.308 | 52.131 |

| | | | | | | | | | |
|---|---|---|---|---|---|---|---|---|---|
| | sd | 0.309$^{e}$ | 0.467$^{f}$ | 0.416$^{g}$ | 0.085$^{b}$ | 0.233$^{cd}$ | 0.104$^{f}$ | 0.593$^{l}$ | 0.586$^{i}$ |
| | RSD | 1.270 | 1.730 | 1.452 | 0.448 | 1.046$^{c}$ | 0.396 | 1.138 | 0.884 |
| Fru | avg | 10.233 | 12.356 | 14.653 | 11.325 | 13.989 | 16.856 | 2.547 | 2.106 |
| | sd | 0.340$^{e}$ | 0.494$^{g}$ | 0.388$^{h}$ | 0.450$^{f}$ | 0.718$^{h}$ | 0.176$^{i}$ | 0.057$^{a}$ | 0.048$^{a}$ |
| | RSD | 3.326 | 3.996 | 2.650 | 3.970 | 5.136 | 1.046 | 2.228 | 2.290 |
| Sac | avg | 2.456 | 3.114 | 4.325 | 1.041 | 2.653 | 2.986 | 0.423 | 0.314 |
| | sd | 0.051$^{c}$ | 0.064$^{d}$ | 0.136$^{e}$ | 0.023$^{b}$ | 0.070$^{cd}$ | 0.059$^{d}$ | 0.036$^{a}$ | 0.010$^{a}$ |
| | RSD | 2.064 | 2.046 | 3.136 | 2.228 | 2.652 | 1.978 | 2.542 | 3.114 |
| Tur | avg | 1.124 | 1.325 | 1.563 | 1.087 | 1.986 | 2.966 | 1.635 | 1.828 |
| | sd | 0.014$^{ab}$ | 0.036$^{bc}$ | 0.062$^{cd}$ | 0.034$^{ab}$ | 0.071$^{e}$ | 0.118$^{f}$ | 0.028$^{cd}$ | 0.056$^{de}$ |
| | RSD | 1.216 | 2.747 | 3.956 | 3.132 | 3.559 | 3.976 | 1.700 | 3.060 |
| Gly | avg | 2.321 | 2.014 | 2.584 | 1.563 | 1.996 | 2.147 | 1.229 | 1.239 |
| | sd | 0.103$^{ef}$ | 0.107$^{de}$ | 0.104$^{f}$ | 0.086$^{c}$ | 0.130$^{d}$ | 0.147$^{de}$ | 0.038$^{b}$ | 0.047$^{b}$ |
| | RSD | 4.428 | 5.306 | 4.024 | 5.528 | 6.534 | 6.845 | 3.087 | 3.830 |
| Galk | avg | 1.653 | 1.896 | 1.993 | 1.815 | 1.963 | 2.056 | 1.457 | 1.482 |
| | sd | 0.025$^{cd}$ | 0.032$^{ef}$ | 0.003$^{ef}$ | 0.079$^{de}$ | 0.078$^{ef}$ | 0.063$^{f}$ | 0.063$^{b}$ | 0.102$^{bc}$ |
| | RSD | 1.494 | 1.688 | 0.143 | 4.340 | 3.978 | 3.087 | 4.332 | 6.895 |
| Gal | avg | 4.325 | 3.586 | 4.856 | 2.325 | 3.856 | 4.658 | 3.554 | 4.660 |
| | sd | 0.261$^{gh}$ | 0.241$^{ef}$ | 0.291$^{h}$ | 0.080$^{c}$ | 0.172$^{fg}$ | 0.153$^{h}$ | 0.093$^{ef}$ | 0.073$^{h}$ |
| | RSD | 6.042 | 6.730 | 5.996 | 3.452 | 4.456 | 3.288 | 2.612 | 1.556 |
| Rib | avg | 0.986 | 1.124 | 1.365 | 1.312 | 2.523 | 3.411 | 1.282 | 1.459 |
| | sd | 0.031$^{bc}$ | 0.037$^{cde}$ | 0.073$^{ef}$ | 0.061d$^{ef}$ | 0.163$^{h}$ | 0.122$^{l}$ | 0.077$^{def}$ | 0.077$^{f}$ |
| | RSD | 3.124 | 3.326 | 5.332 | 4.644 | 6.446 | 3.584 | 5.996 | 5.306 |
| iMAL | avg | 0.008 | 0.007 | 1.241 | 0.495 | 0.459 | 0.663 | 1.412 | 1.580 |
| | sd | 0.000$^{a}$ | 0.000$^{a}$ | 0.059$^{g}$ | 0.020$^{c}$ | 0.030$^{c}$ | 0.036$^{d}$ | 0.030$^{h}$ | 0.030$^{i}$ |
| | RSD | 3.963 | 4.726 | 4.730 | 4.112 | 6.506 | 5.370 | 2.156 | 1.896 |
| iMALT | avg | 0.009 | 0.010 | 0.015 | 0.563 | 0.689 | 0.852 | 0.786 | 0.774 |
| | sd | 0.000$^{a}$ | 0.000$^{a}$ | 0.000$^{a}$ | 0.001$^{b}$ | 0.004$^{de}$ | 0.002$^{f}$ | 0.004$^{f}$ | 0.009$^{ef}$ |
| | RSD | 0.378 | 1.812 | 1.136 | 0.224 | 0.536 | 0.242 | 0.482 | 1.108 |
| Mal | avg | 1.245 | 1.235 | 1.523 | 0.293 | 0.452 | 0.896 | 2.005 | 1.998 |
| | sd | 0.031$^{de}$ | 0.038$^{de}$ | 0.034$^{e}$ | 0.015$^{a}$ | 0.031$^{ab}$ | 0.066$^{cd}$ | 0.187$^{f}$ | 0.240$^{f}$ |
| | RSD | 2.470 | 3.084 | 2.228 | 5.102 | 6.830 | 7.316 | 9.306 | 11.992 |
| MALt | avg | 0.005 | 0.007 | 0.011 | 0.008 | 0.009 | 0.009 | 3.554 | 2.638 |
| | sd | 0.000$^{a}$ | 0.000$^{a}$ | 0.000$^{a}$ | 0.000$^{a}$ | 0.000$^{a}$ | 0.000$^{a}$ | 0.182$^{f}$ | 0.172$^{d}$ |
| | RSD | 2.650 | 1.556 | 1.272 | 3.970 | 3.242 | 3.624 | 5.126 | 6.512 |
| Mant | avg | 0.896 | 0.925 | 0.986 | 0.852 | 0.963 | 1.042 | 11.539 | 10.332 |
| | sd | 0.020$^{ab}$ | 0.023$^{ab}$ | 0.021$^{ab}$ | 0.005$^{a}$ | 0.009$^{ab}$ | 0.006$^{b}$ | 0.023$^{i}$ | 0.018$^{h}$ |
| | RSD | 2.224 | 2.516 | 2.170 | 0.616 | 0.904 | 0.596 | 0.202 | 0.178 |
| Xyl | avg | 1.023 | 1.234 | 1.425 | 0.885 | 0.996 | 1.147 | 0.942 | 0.947 |
| | sd | 0.002$^{f}$ | 0.002$^{h}$ | 0.047$^{i}$ | 0.001$^{cd}$ | 0.001$^{ef}$ | 0.000$^{g}$ | 0.025$^{de}$ | 0.031$^{de}$ |
| | RSD | 0.178 | 0.136 | 3.316 | 0.084 | 0.112 | 0.024 | 2.664 | 3.306 |
| Man | avg | 0.031 | 0.042 | 0.058 | 0.029 | 0.041 | 0.063 | 1.323 | 1.312 |
| | sd | 0.001$^{a}$ | 0.002$^{a}$ | 0.003$^{a}$ | 0.001$^{a}$ | 0.002$^{a}$ | 0.002$^{a}$ | 0.039$^{d}$ | 0.028$^{d}$ |
| | RSD | 2.066 | 3.970 | 5.578 | 4.130 | 4.642 | 3.792 | 2.985 | 2.145 |
| Pan | avg | 0.010 | 0.012 | 0.018 | 0.008 | 0.012 | 0.015 | 0.563 | 0.589 |
| | sd | 0.000$^{a}$ | 0.000$^{a}$ | 0.000$^{a}$ | 0.000$^{a}$ | 0.001$^{a}$ | 0.000$^{a}$ | 0.021$^{cd}$ | 0.025$^{de}$ |
| | RSD | 1.206 | 2.058 | 1.761 | 3.163 | 4.478 | 2.011 | 3.706 | 4.318 |
| Ram | avg | 1.025 | 1.235 | 1.412 | 0.856 | 0.963 | 1.023 | 0.325 | 0.355 |
| | sd | 0.036$^{ef}$ | 0.053$^{g}$ | 0.064$^{h}$ | 0.023$^{d}$ | 0.035$^{e}$ | 0.027$^{ef}$ | 0.001$^{b}$ | 0.001$^{b}$ |

| | | | | | | | | | |
|---|---|---|---|---|---|---|---|---|---|
| | RSD | 2.721 | 3.662 | 2.616 | 3.518 | 4.332 | 4.507 | 0.316 | 0.376 |
| Raf | avg | 1.325 | 1.452 | 1.568 | 0.752 | 0.863 | 0.996 | 2.426 | 2.410 |
| | sd | $0.022^{c}$ | $0.014^{d}$ | $0.012^{d}$ | $0.006^{a}$ | $0.006^{a}$ | $0.036^{b}$ | $0.053^{g}$ | $0.081^{g}$ |
| | RSD | 1.663 | 0.948 | 0.776 | 0.762 | 0.666 | 3.613 | 2.166 | 3.350 |
| Sth | avg | 0.563 | 0.633 | 0.741 | 0.114 | 0.358 | 0.552 | 0.556 | 0.561 |
| | sd | $0.007^{c}$ | $0.012^{d}$ | $0.007^{e}$ | $0.003^{a}$ | $0.015^{b}$ | $0.011^{c}$ | $0.000^{c}$ | $0.000^{c}$ |
| | RSD | 1.163 | 1.948 | 1.006 | 2.329 | 4.316 | 1.948 | 0.089 | 0.062 |

Legend: Mean of three replicates ± standard deviation. Different letters in the same row indicate statistically significant difference at $p < 0.05$. RSD (relative standard deviation). Abbreviations: IM - Mongolian Chaga, MW 96% EtOH, 50% EtOH, $H_2O$ - microwave-assisted extraction, VAE 96% EtOH, 50% EtOH, $H_2O$ - ultrasound-assisted extraction, SWE 200 °C, 120 °C – subcritical water extraction; Sor - Sorbitol; Tre - trehalose; Ara - arabinose ; Glu - glucose; Fru - fructose; Sac - saccharose; Tur - turanose; Gly - Glycerol; Galk - galactitol; Gal - galactose; Rib - ribose; iMAL - isomaltose; iMALt isomaltotriosea; Mal - maltose; MALt - maltotriose; Mant - mannitol; Xyl - xylose; Man-mannose; Pan - panose; Ram - ramnose; Raf -raffinose; Sth - stachyose.

**Table S3.** Polyphenol profile of Serbian *I. obliquus* extracts obtained by different green extraction techniques (mg/kg).

| **mg/kg** | | **IS-MW 96% ETOH** | **IS-MW 50% ETOH** | **IS-MW water** | **IS-VAE 96% ETOH** | **IS-VAE 50% ETOH** | **IS-VAE water** | **IS-SWE 200°C** | **IS-SWE 120°C** |
|---|---|---|---|---|---|---|---|---|---|
| Protocatechuic_acid | avg | 0.99 | 0.97 | 0.83 | 1.24 | 1.31 | 0.98 | 0.99 | 1.49 |
| | sd | $0.01^{d}$ | $0.01^{d}$ | $0.01^{b}$ | $0.01^{f}$ | $0.02^{g}$ | $0.01^{d}$ | $0.00^{d}$ | $0.00^{h}$ |
| | RSD | 1.00 | 0.89 | 1.11 | 1.04 | 1.33 | 1.21 | 1.16 | 1.43 |
| Chlorogenic acid | avg | 812.32 | 897.32 | 741.27 | 795.32 | 845.36 | 658.63 | 840.35 | 697.42 |
| | sd | $0.12^{h}$ | $0.16^{l}$ | $0.21^{d}$ | $0.16^{f}$ | $0.20^{k}$ | $0.22^{b}$ | $1.09^{j}$ | $0.93^{c}$ |
| | RSD | 0.95 | 1.45 | 1.55 | 1.24 | 1.69 | 1.46 | 9.12 | 6.5 |
| p-Hydroxybenzoic acid | avg | 0.33 | 0.38 | 0.15 | 0.51 | 0.47 | 0.39 | 0.23 | 0.55 |
| | sd | $0.00^{f}$ | $0.00^{g}$ | $0.00^{a}$ | $0.00^{j}$ | $0.00^{i}$ | $0.00^{gh}$ | $0.01^{c}$ | $0.01^{k}$ |
| | RSD | 0.13 | 0.04 | 0.04 | 0.11 | 0.14 | 0.31 | 1.65 | 1.03 |
| Catechin | avg | 2.80 | 2.99 | 2.94 | 3.49 | 3.39 | 2.99 | 1.80 | 2.41 |
| | sd | $0.02^{d}$ | $0.03^{b}$ | $0.08^{d}$ | $0.04^{e}$ | $0.07^{e}$ | $0.03^{d}$ | $0.05^{b}$ | $0.10^{c}$ |
| | RSD | 0.88 | 1.42 | 2.82 | 1.11 | 1.99 | 1.02 | 2.86 | 4.01 |
| p-Hydroxyphenylacetic_acid | avg | 0.11 | 0.14 | 0.15 | 0.15 | 0.13 | 0.11 | 0.11 | 0.12 |
| | sd | 0.00 | 0.00 | 0.00 | 0.00 | 0.00 | 0.00 | 0.00 | 0.00 |
| | RSD | 0.70 | 0.40 | 0.43 | 0.87 | 0.91 | 0.63 | 0.66 | 0.92 |
| Caffeic acid | avg | 0.84 | 0.85 | 0.78 | 0.94 | 0.88 | 0.76 | 0.98 | 2.66 |
| | sd | $0.02^{ab}$ | $0.03^{ab}$ | $0.06^{a}$ | $0.04^{abc}$ | $0.04^{ab}$ | $0.03^{a}$ | $0.04^{bc}$ | $0.14^{f}$ |
| | RSD | 2.05 | 3.99 | 2.86 | 3.73 | 4.91 | 4.02 | 4.08 | 5.36 |
| Vanillic acid | avg | 0.27 | 0.29 | 0.42 | 0.27 | 0.29 | 0.24 | 0.27 | 0.44 |
| | sd | $0.00^{c}$ | $0.00^{e}$ | $0.00^{i}$ | $0.00^{c}$ | $0.00^{e}$ | $0.01^{a}$ | $0.00^{c}$ | $0.00^{j}$ |
| | RSD | 0.16 | 0.23 | 0.15 | 1.27 | 1.75 | 2.28 | 0.31 | 0.39 |
| Syringic acid | avg | 0.12 | 0.14 | 0.23 | 0.52 | 0.52 | 0.50 | 0.13 | 0.11 |
| | sd | $0.00^{bc}$ | $0.00^{d}$ | $0.00^{e}$ | $0.00^{k}$ | $0.00^{k}$ | $0.00^{j}$ | $0.00^{cd}$ | $0.00^{b}$ |
| | RSD | 0.47 | 0.13 | 0.11 | 0.37 | 0.25 | 0.61 | 3.79 | 2.82 |
| Rutin | avg | 0.10 | 0.10 | 0.08 | 0.08 | 0.09 | 0.08 | 0.09 | 0.07 |
| | sd | $0.00^{d}$ | $0.00^{d}$ | $0.00^{b}$ | $0.00^{f}$ | $0.01^{c}$ | $0.00^{b}$ | $0.00^{c}$ | $0.00^{a}$ |
| | RSD | 1.05 | 1.37 | 0.97 | 1.55 | 2.06 | 1.48 | 4.11 | 5.02 |
| | avg | 0.41 | 0.45 | 0.59 | 0.24 | 0.28 | 0.23 | 1.35 | 1.22 |

| | | | | | | | | | |
|---|---|---|---|---|---|---|---|---|---|
| Quercetin 3-O-glucoside | sd | $0.01^{d}$ | $0.00^{e}$ | $0.01^{g}$ | $0.01^{a}$ | $0.01^{b}$ | $0.01^{a}$ | $0.00^{i}$ | $0.01^{h}$ |
| | RSD | 1.38 | 1.10 | 0.54 | 2.23 | 3.31 | 3.56 | 0.46 | 0.89 |
| p-Coumaric_acid | avg | 1.42 | 1.59 | 1.10 | 1.46 | 1.41 | 1.40 | 1.55 | 3.07 |
| | sd | $0.02^{f}$ | $0.04^{gh}$ | $0.02^{c}$ | $0.00^{f}$ | $0.00^{f}$ | $0.00^{ef}$ | $0.01^{g}$ | $0.04^{i}$ |
| | RSD | 1.49 | 2.31 | 2.02 | 0.65 | 1.05 | 0.37 | 0.86 | 1.20 |
| Isorhamnetin_3-O-rutinoside | avg | 0.12 | 0.13 | 0.13 | 1.12 | 1.06 | 0.98 | 0.11 | 0.11 |
| | sd | $0.00^{a}$ | $0.00^{a}$ | $0.00^{a}$ | $0.01^{i}$ | $0.01^{h}$ | $0.02^{f}$ | $0.00^{a}$ | $0.00^{a}$ |
| | RSD | 2.43 | 3.26 | 2.24 | 0.82 | 1.02 | 1.60 | 1.00 | 1.38 |
| Sinapic_acid | avg | 0.10 | 0.10 | 0.11 | 0.10 | 0.11 | 0.10 | 0.11 | 0.12 |
| | sd | $0.00^{a}$ | $0.00^{a}$ | $0.00^{ab}$ | $0.00^{a}$ | $0.00^{ab}$ | $0.00^{ab}$ | $0.00^{ab}$ | $0.00^{b}$ |
| | RSD | 0.52 | 1.27 | 1.24 | 1.19 | 0.85 | 0.82 | 1.97 | 1.14 |
| Ferulic_acid | avg | 0.26 | 0.27 | 0.63 | 0.23 | 0.29 | 0.28 | 0.29 | 0.47 |
| | sd | $0.01^{cd}$ | $0.01^{d}$ | $0.02^{h}$ | $0.00^{bc}$ | $0.00^{d}$ | $0.00^{d}$ | $0.01^{d}$ | $0.01^{g}$ |
| | RSD | 3.51 | 4.45 | 3.36 | 0.98 | 1.58 | 0.85 | 3.21 | 2.55 |
| Cinnamic acid | avg | 4.33 | 4.56 | 5.69 | 2.65 | 4.52 | 1.87 | 4.33 | 8.94 |
| | sd | $0.13^{c}$ | $0.15^{cd}$ | $0.23^{efg}$ | $0.02^{b}$ | $0.08^{cd}$ | $0.06^{a}$ | $0.24^{c}$ | $0.74^{i}$ |
| | RSD | 2.97 | 3.22 | 3.99 | 0.64 | 1.85 | 3.23 | 5.62 | 8.24 |
| Quercetin 3-O-rhamnoside | avg | 0.33 | 0.33 | 0.14 | 0.09 | 0.09 | 0.08 | 4.47 | 4.06 |
| | sd | $0.01^{ab}$ | $0.05^{ab}$ | $0.01^{ab}$ | $0.00^{a}$ | $0.00^{a}$ | $0.00^{a}$ | $0.22^{f}$ | $0.26^{e}$ |
| | RSD | 0.52 | 2.12 | 0.97 | 1.17 | 2.15 | 1.86 | 5.02 | 6.32 |
| Kaempferol_7-O-glucoside | avg | 0.06 | 0.08 | 0.06 | 0.04 | 0.05 | 0.04 | 1.53 | 1.49 |
| | sd | $0.05^{a}$ | $0.07^{a}$ | $0.00^{a}$ | $0.00^{a}$ | $0.00^{a}$ | $0.00^{a}$ | $0.05^{c}$ | $0.06^{c}$ |
| | RSD | 2.90 | 3.78 | 2.77 | 3.53 | 4.11 | 5.05 | 3.21 | 5.26 |
| Phlorizin | avg | 0.83 | 0.87 | 0.54 | 0.40 | 0.44 | 0.39 | 0.78 | 0.36 |
| | sd | $0.02^{cd}$ | $0.00^{d}$ | $0.00^{b}$ | $0.01^{a}$ | $0.01^{a}$ | $0.00^{a}$ | $0.02^{c}$ | $0.01^{a}$ |
| | RSD | 2.00 | 0.30 | 0.27 | 1.58 | 1.33 | 1.26 | 2.69 | 1.77 |
| Eriodictyol | avg | 0.06 | 0.06 | 0.03 | 0.23 | 0.21 | 0.20 | 0.03 | 0.05 |
| | sd | $0.00^{b}$ | $0.00^{b}$ | $0.00^{a}$ | $0.01^{d}$ | $0.01^{c}$ | $0.00^{c}$ | $0.00^{a}$ | $0.00^{b}$ |
| | RSD | 3.25 | 3.90 | 3.09 | 2.46 | 4.66 | 2.02 | 0.65 | 1.03 |
| Quercetin | avg | 1.45 | 1.65 | 1.75 | 1.25 | 1.46 | 1.10 | 0.34 | 0.49 |
| | sd | $0.02^{de}$ | $0.04^{ef}$ | $0.03^{ef}$ | $0.04^{cd}$ | $0.02^{de}$ | $0.03^{c}$ | $0.04^{a}$ | $0.05^{ab}$ |
| | RSD | 1.12 | 2.63 | 4.33 | 3.26 | 1.33 | 2.65 | 1.95 | 2.35 |
| Phloretin | avg | 0.02 | 0.04 | 0.03 | 0.06 | 0.07 | 0.05 | 0.02 | 0.04 |
| | sd | 0.00 | 0.00 | 0.00 | 0.00 | 0.00 | 0.00 | 0.00 | 0.00 |
| | RSD | 4.93 | 4.78 | 2.58 | 2.29 | 2.55 | 1.35 | 0.58 | 0.68 |
| Naringenin | avg | 0.02 | 0.03 | 0.02 | 0.03 | 0.04 | 0.03 | 0.02 | 0.02 |
| | sd | 0.00 | 0.00 | 0.00 | 0.00 | 0.00 | 0.00 | 0.00 | 0.00 |
| | RSD | 1.01 | 1.24 | 0.95 | 0.76 | 0.90 | 0.94 | 0.45 | 0.50 |
| Gallic_acid | avg | 1.26 | 1.57 | 1.00 | 2.15 | 2.56 | 1.87 | 0.42 | 0.13 |
| | sd | $0.01^{f}$ | $0.01^{h}$ | $0.00^{d}$ | $0.01^{k}$ | $0.01^{m}$ | $0.01^{i}$ | $0.01^{c}$ | $0.01^{a}$ |
| | RSD | 0.44 | 0.35 | 0.33 | 0.47 | 0.45 | 0.62 | 0.44 | 0.46 |

Legend: The results are expressed as mean values of three replicates ± standard deviation. Different letters in the same row indicate statistically significant difference at $p < 0.05$. Abbreviations: IS - Serbian Chaga, MW 96% EtOH, 50% EtOH, $H_2O$ - microwave-assisted extraction, VAE 96% EtOH, 50% EtOH, $H_2O$ - ultrasound-assisted extraction, SWE 200 °C, 120 °C.

**Table S4.** Polyphenol profile of Mongolian *I. obliquus* extracts obtained by different green extraction techniques (mg/kg).

| mg/kg | | IM-MW 96% ETOH | IM-MW 50% ETOH | IM-MW water | IM-VAE 96% ETOH | IM-VAE 50% ETOH | IM-VAE water | IM-SWE 200º°C | IM-SWE 120°C |
|---|---|---|---|---|---|---|---|---|---|
| Protocatechuic acid | avg | 1.03 | 1.26 | 0.97 | 0.85 | 0.91 | 0.79 | 0.83 | 1.25 |
| | sd | 0.00$^{e}$ | 0.00$^{f}$ | 0.00$^{d}$ | 0.00$^{b}$ | 0.00$^{c}$ | 0.00$^{a}$ | 0.00$^{b}$ | 0.00$^{f}$ |
| | RSD | 0.09 | 0.24 | 0.30 | 0.18 | 0.23 | 0.21 | 0.21 | 0.36 |
| Chlorogenic acid | avg | 825.32 | 798.63 | 897.32 | 742.63 | 768.96 | 642.12 | 741.27 | 970.56 |
| | sd | 0.24$^{i}$ | 0.32$^{g}$ | 0.21$^{l}$ | 0.63$^{d}$ | 0.73$^{e}$ | 1.28$^{a}$ | 1.23$^{d}$ | 0.89$^{m}$ |
| | RSD | 1.95 | 2.56 | 1.9 | 4.64 | 5.58 | 8.21 | 9.14 | 8.66 |
| p-Hydroxybenzoic acid | avg | 0.30 | 0.32 | 0.28 | 0.38 | 0.41 | 0.29 | 0.15 | 0.20 |
| | sd | 0.00$^{de}$ | 0.00$^{ef}$ | 0.01$^{d}$ | 0.00$^{g}$ | 0.02$^{h}$ | 0.01$^{d}$ | 0.00$^{a}$ | 0.00$^{b}$ |
| | RSD | 0.33 | 1.14 | 1.82 | 1.24 | 4.65 | 2.78 | 0.98 | 1.13 |
| Catechin | avg | 5.99 | 5.24 | 4.99 | 5.76 | 6.10 | 4.33 | 2.94 | 1.51 |
| | sd | 0.06$^{hi}$ | 0.07$^{g}$ | 0.04$^{g}$ | 0.15$^{h}$ | 0.12$^{i}$ | 0.20$^{f}$ | 0.07$^{d}$ | 0.05a |
| | RSD | 1.00 | 1.26 | 0.90 | 2.65 | 2.01 | 4.59 | 2.22 | 3.25 |
| p-Hydroxy-phenylaceticacid | avg | 0.15 | 0.18 | 0.14 | 0.13 | 0.14 | 0.11 | 0.15 | 0.07 |
| | sd | 0.00 | 0.00 | 0.00 | 0.00 | 0.00 | 0.00 | 0.00 | 0.00 |
| | RSD | 0.68 | 1.26 | 2.01 | 1.39 | 2.37 | 3.11 | 0.80 | 0.82 |
| Caffeic acid | avg | 4.01 | 4.26 | 3.97 | 2.05 | 2.53 | 1.86 | 2.24 | 1.13 |
| | sd | 0.03$^{g}$ | 0.16$^{h}$ | 0.09$^{g}$ | 0.01$^{de}$ | 0.02$^{f}$ | 0.02$^{d}$ | 0.09$^{e}$ | 0.01$^{c}$ |
| | RSD | 0.71 | 3.77 | 2.23 | 0.49 | 0.92 | 1.26 | 4.10 | 1.33 |
| Vanillic acid | avg | 0.33 | 0.36 | 0.28 | 0.25 | 0.29 | 0.24 | 0.42 | 0.38 |
| | sd | 0.00$^{f}$ | 0.00$^{g}$ | 0.00$^{d}$ | 0.00$^{b}$ | 0.00$^{e}$ | 0.00$^{a}$ | 0.00$^{i}$ | 0.00$^{h}$ |
| | RSD | 0.38 | 0.47 | 0.35 | 1.02 | 1.71 | 1.35 | 0.28 | 0.53 |
| Syringic acid | avg | 0.39 | 0.41 | 0.31 | 0.36 | 0.41 | 0.30 | 0.23 | 0.04 |
| | sd | 0.00$^{h}$ | 0.00$^{i}$ | 0.00$^{f}$ | 0.00$^{g}$ | 0.01$^{i}$ | 0.01$^{f}$ | 0.01$^{e}$ | 0.00$^{a}$ |
| | RSD | 0.62 | 0.58 | 0.61 | 0.58 | 2.25 | 1.99 | 2.85 | 3.97 |
| Rutin | avg | 0.35 | 0.46 | 0.30 | 0.30 | 0.36 | 0.28 | 0.28 | 0.24 |
| | sd | 0.00$^{h}$ | 0.00$^{j}$ | 0.00$^{g}$ | 0.00$^{g}$ | 0.00i | 0.01$^{f}$ | 0.00$^{f}$ | 0.00$^{e}$ |
| | RSD | 0.16 | 0.21 | 0.18 | 1.11 | 1.00 | 3.13 | 4.12 | 3.24 |
| Quercetin 3-O-glu-coside | avg | 0.46 | 0.53 | 0.34 | 0.29 | 0.31 | 0.24 | 1.59 | 1.42 |
| | sd | 0.02$^{e}$ | 0.01$^{f}$ | 0.01$^{c}$ | 0.01$^{b}$ | 0.01$^{bc}$ | 0.01$^{a}$ | 0.01$^{j}$ | 0.01$^{i}$ |
| | RSD | 3.33 | 2.46 | 2.90 | 3.11 | 4.03 | 3.58 | 0.77 | 0.76 |
| p-Coumaric_acid | avg | 1.24 | 1.66 | 0.99 | 1.43 | 1.56 | 1.33 | 1.10 | 0.85 |
| | sd | 0.03$^{d}$ | 0.05$^{h}$ | 0.01$^{b}$ | 0.02$^{f}$ | 0.03$^{g}$ | 0.03$^{e}$ | 0.01$^{c}$ | 0.01$^{a}$ |
| | RSD | 2.28 | 3.12 | 1.51 | 1.75 | 2.11 | 2.47 | 0.82 | 1.31 |
| Isorhamnetin 3-O-rutinoside | avg | 1.01 | 1.36 | 0.89 | 0.39 | 0.43 | 0.29 | 0.13 | 0.12 |
| | sd | 0.00$^{g}$ | 0.00$^{j}$ | 0.00$^{e}$ | 0.01$^{c}$ | 0.01$^{d}$ | 0.01$^{b}$ | 0.00$^{a}$ | 0.00$^{a}$ |
| | RSD | 0.23 | 0.28 | 0.22 | 2.65 | 3.32 | 2.85 | 0.89 | 1.79 |
| Sinapic acid | avg | 0.43 | 0.65 | 0.16 | 0.25 | 0.36 | 0.20 | 0.27 | 0.26 |
| | sd | 0.01$^{h}$ | 0.01$^{i}$ | 0.01$^{de}$ | 0.00$^{d}$ | 0.01$^{f}$ | 0.00$^{c}$ | 0.00$^{e}$ | 0.00$^{de}$ |
| | RSD | 0.73 | 0.72 | 0.81 | 0.77 | 3.16 | 1.79 | 0.90 | 1.32 |
| Ferulic acid | avg | 0.43 | 0.67 | 0.23 | 0.21 | 0.35 | 0.19 | 0.63 | 0.64 |
| | sd | 0.01$^{f}$ | 0.02$^{i}$ | 0.01$^{bc}$ | 0.01$^{ab}$ | 0.02$^{e}$ | 0.00$^{a}$ | 0.02$^{h}$ | 0.02$^{h}$ |
| | RSD | 2.25 | 3.38 | 2.85 | 6.52 | 5.79 | 1.33 | 3.14 | 3.85 |
| Cinnamic acid | avg | 5.21 | 5.66 | 4.21 | 6.33 | 7.00 | 5.87 | 5.69 | 6.43 |
| | sd | 0.12$^{de}$ | 0.22$^{ef}$ | 0.17$^{c}$ | 0.18$^{fgh}$ | 0.07$^{h}$ | 0.11$^{efg}$ | 0.34$^{efg}$ | 0.09$^{gh}$ |
| | RSD | 2.24 | 3.86 | 4.11 | 2.89 | 1.05 | 1.89 | 5.99 | 1.33 |

| | | | | | | | | | |
|---|---|---|---|---|---|---|---|---|---|
| Quercetin 3-O-rhamnoside | avg | 0.31 | 0.36 | 0.20 | 0.08 | 0.08 | 0.07 | 1.14 | 1.88 |
| | sd | 0.01$^{ab}$ | 0.01$^{b}$ | 0.00$^{ab}$ | 0.00$^{a}$ | 0.00$^{a}$ | 0.00$^{a}$ | 0.05$^{c}$ | 0.06$^{d}$ |
| | RSD | 1.87 | 2.99 | 1.75 | 2.00 | 2.69 | 3.05 | 4.77 | 3.00 |
| Kaempferol_7-O-glucoside | avg | 0.09 | 0.11 | 0.04 | 0.01 | 0.02 | 0.01 | 1.33 | 1.51 |
| | sd | 0.00$^{a}$ | 0.00$^{a}$ | 0.00$^{a}$ | 0.00$^{a}$ | 0.00$^{a}$ | 0.00$^{a}$ | 0.04$^{b}$ | 0.13$^{c}$ |
| | RSD | 1.79 | 2.37 | 1.63 | 3.12 | 4.33 | 3.90 | 3.00 | 8.45 |
| Phlorizin | avg | 1.64 | 1.76 | 1.43 | 1.40 | 1.46 | 1.30 | 1.54 | 1.22 |
| | sd | 0.02$^{h}$ | 0.07$^{i}$ | 0.07$^{f}$ | 0.02$^{f}$ | 0.01$^{fg}$ | 0.00$^{e}$ | 0.02$^{g}$ | 0.00$^{e}$ |
| | RSD | 1.22 | 3.99 | 5.25 | 4.25 | 1.52 | 0.85 | 1.21 | 1.57 |
| Eriodictyol | avg | 0.49 | 0.52 | 0.41 | 0.05 | 0.05 | 0.05 | 0.75 | 0.65 |
| | sd | 0.01$^{f}$ | 0.01$^{g}$ | 0.00$^{e}$ | 0.00$^{b}$ | 0.00$^{b}$ | 0.00$^{b}$ | 0.00$^{i}$ | 0.00$^{h}$ |
| | RSD | 1.33 | 1.65 | 1.11 | 2.77 | 2.65 | 1.86 | 0.66 | 0.65 |
| Quercetin | avg | 4.33 | 6.85 | 2.33 | 2.11 | 2.56 | 1.85 | 0.75 | 0.65 |
| | sd | 0.16$^{j}$ | 0.27$^{k}$ | 0.04$^{hi}$ | 0.05$^{gh}$ | 0.09$^{i}$ | 0.05$^{fg}$ | 0.05$^{b}$ | 0.06$^{b}$ |
| | RSD | 3.68 | 4.01 | 1.89 | 2.35 | 3.37 | 2.86 | 2.88 | 3.11 |
| Phloretin | avg | 0.07 | 0.10 | 0.04 | 0.07 | 0.07 | 0.06 | 0.07 | 0.05 |
| | sd | 0.00 | 0.00 | 0.00 | 0.00 | 0.00 | 0.00 | 0.00 | 0.00 |
| | RSD | 1.29 | 2.00 | 1.13 | 0.53 | 0.83 | 0.87 | 0.57 | 1.87 |
| Naringenin | avg | 0.04 | 0.05 | 0.02 | 0.05 | 0.06 | 0.05 | 0.02 | 0.05 |
| | sd | 0.00 | 0.00 | 0.00 | 0.00 | 0.00 | 0.00 | 0.00 | 0.00 |
| | RSD | 0.39 | 0.40 | 0.37 | 0.52 | 0.62 | 0.54 | 0.43 | 0.65 |
| Gallic_acid | avg | 1.14 | 1.43 | 1.32 | 2.33 | 2.69 | 2.00 | 0.34 | 0.40 |
| | sd | 0.01$^{e}$ | 0.01$^{g}$ | 0.01$^{f}$ | 0.02$^{l}$ | 0.08$^{n}$ | 0.05$^{j}$ | 0.00$^{b}$ | 0.00$^{bc}$ |
| | RSD | 0.63 | 0.68 | 0.83 | 0.75 | 2.90 | 2.29 | 2.11 | 2.86 |

Legend: The results are expressed as mean values of three replicates ± standard deviation. Different letters in the same row indicate statistically significant difference at $p < 0.05$. RSD (relative standard deviation) Abbreviations: IS - Serbian Chaga, MW 96% EtOH, 50% EtOH, $H_2O$ - microwave-assisted extraction, VAE 96% EtOH, 50% EtOH, $H_2O$ - ultrasound-assisted extraction, SWE 200 °C, 120 °C.

**Table S5.** Fatty acid profile of Serbian *I. obliquus* extracts obtained by different green extraction tech-niques mg/100 g.

| **mg/100 g** | | **IS-MW 96% ETOH** | **IS-MW 50% ETOH** | **IS-MW water** | **IS-VAE 96% ETOH** | **IS-VAE 50% ETOH** | **IS-VAE water** | **IS-SWE 200°C** | **IS-SWE 120°C** |
|---|---|---|---|---|---|---|---|---|---|
| C4:0 butyric acid | avg | 0.030 | 0.020 | 0.020 | 0.060 | 0.060 | 0.040 | 0.090 | 0.080 |
| | sd | 0.000$^{b}$ | 0.000$^{a}$ | 0.000$^{a}$ | 0.002$^{e}$ | 0.002$^{e}$ | 0.001$^{c}$ | 0.005$^{g}$ | 0.002$^{f}$ |
| | RSD | 1.178 | 0.777 | 1.073 | 3.166 | 3.663 | 2.163 | 5.166 | 2.166 |
| C6:0 caproic acid | avg | 0.020 | 0.030 | 0.010 | 0.040 | 0.040 | 0.020 | 0.090 | 0.040 |
| | sd | 0.000$^{b}$ | 0.000$^{c}$ | 0.000$^{a}$ | 0.001$^{d}$ | 0.000$^{d}$ | 0.000$^{b}$ | 0.002$^{h}$ | 0.001$^{d}$ |
| | RSD | 1.011 | 1.343 | 1.663 | 1.556 | 1.073 | 0.997 | 2.045 | 3.112 |
| C8:0 caprilic acid | avg | 0.050 | 0.060 | 0.030 | 0.110 | 0.090 | 0.060 | 0.160 | 0.170 |
| | sd | 0.001$^{b}$ | 0.002$^{c}$ | 0.001$^{a}$ | 0.003$^{f}$ | 0.003$^{e}$ | 0.000$^{c}$ | 0.001$^{i}$ | 0.001$^{j}$ |
| | RSD | 2.090 | 3.956 | 2.816 | 2.311 | 3.866 | 0.697 | 0.674 | 0.787 |
| C10:0 capric acid | avg | 0.230 | 0.210 | 0.140 | 0.320 | 0.330 | 0.190 | 0.580 | 0.420 |
| | sd | 0.012$^{bc}$ | 0.013$^{bc}$ | 0.007$^{a}$ | 0.019$^{d}$ | 0.025$^{d}$ | 0.015$^{b}$ | 0.008$^{g}$ | 0.008$^{e}$ |
| | RSD | 5.062 | 6.064 | 4.899 | 5.986 | 7.456 | 8.156 | 1.437 | 1.842 |
| C11:0 undeca-noic acid | avg | 0.110 | 0.100 | 0.070 | 0.160 | 0.150 | 0.060 | 0.650 | 0.320 |
| | sd | 0.001$^{b}$ | 0.001$^{b}$ | 0.000$^{a}$ | 0.006$^{c}$ | 0.005$^{c}$ | 0.002$^{a}$ | 0.015$^{i}$ | 0.013$^{f}$ |
| | RSD | 0.803 | 0.718 | 0.674 | 3.665 | 3.261 | 3.014 | 2.279 | 3.936 |

| | | | | | | | | | |
|---|---|---|---|---|---|---|---|---|---|
| C12:0 lauric acid | avg | 0.120 | 0.120 | 0.040 | 0.110 | 0.130 | 0.060 | 0.420 | 0.190 |
| | sd | 0.001$^{de}$ | 0.001$^{de}$ | 0.000$^{a}$ | 0.001$^{d}$ | 0.001$^{e}$ | 0.000$^{b}$ | 0.000$^{k}$ | 0.000$^{g}$ |
| | RSD | 0.690 | 1.161 | 0.638 | 0.865 | 1.098 | 0.732 | 0.062 | 0.053 |
| C13:0 tride-canoic acid | avg | 0.040 | 0.030 | 0.020 | 0.050 | 0.050 | 0.020 | 0.140 | 0.110 |
| | sd | 0.001$^{bc}$ | 0.000$^{ab}$ | 0.000$^{a}$ | 0.001$^{cd}$ | 0.001$^{cd}$ | 0.000$^{a}$ | 0.005$^{h}$ | 0.003$^{g}$ |
| | RSD | 1.479 | 0.547 | 1.861 | 2.027 | 2.255 | 0.552 | 3.725 | 3.032 |
| C14:0 myristic acid | avg | 0.660 | 0.690 | 0.230 | 0.740 | 0.720 | 0.330 | 0.930 | 0.620 |
| | sd | 0.018$^{ef}$ | 0.017$^{fg}$ | 0.003$^{a}$ | 0.010$^{h}$ | 0.015$^{gh}$ | 0.005$^{b}$ | 0.019$^{j}$ | 0.015$^{e}$ |
| | RSD | 2.790 | 2.468 | 1.416 | 1.376 | 2.097 | 1.651 | 2.033 | 2.411 |
| C14:1 myristoleic acid | avg | 0.580 | 0.510 | 0.320 | 0.670 | 0.640 | 0.330 | 0.650 | 0.410 |
| | sd | 0.003$^{g}$ | 0.016$^{f}$ | 0.007$^{b}$ | 0.016$^{i}$ | 0.018$^{h}$ | 0.001$^{b}$ | 0.008$^{h}$ | 0.007$^{c}$ |
| | RSD | 0.442 | 3.124 | 2.044 | 2.442 | 2.852 | 0.304 | 1.224 | 1.792 |
| C15:0 pentadeca-noic acid | avg | 0.870 | 0.860 | 0.330 | 0.960 | 1.030 | 0.420 | 1.450 | 1.230 |
| | sd | 0.013$^{d}$ | 0.020$^{d}$ | 0.005$^{a}$ | 0.011$^{e}$ | 0.017$^{g}$ | 0.008$^{b}$ | 0.008$^{k}$ | 0.010$^{j}$ |
| | RSD | 1.548 | 2.330 | 1.484 | 1.182 | 1.628 | 1.990 | 0.536 | 0.842 |
| C15:1 pen-tadesenoic acid | avg | 0.060 | 0.050 | 0.020 | 0.030 | 0.020 | 0.010 | 0.120 | 0.080 |
| | sd | 0.000$^{e}$ | 0.000$^{d}$ | 0.000$^{b}$ | 0.000$^{c}$ | 0.000$^{b}$ | 0.000$^{a}$ | 0.001$^{h}$ | 0.001$^{g}$ |
| | RSD | 0.664 | 0.712 | 0.534 | 1.136 | 1.198 | 0.764 | 1.148 | 1.570 |
| C16:0 palmitic acid | avg | 18.320 | 16.220 | 15.660 | 19.320 | 18.880 | 14.320 | 18.650 | 16.520 |
| | sd | 0.122$^{e}$ | 0.149$^{d}$ | 0.101$^{cd}$ | 0.318$^{e}$ | 0.411$^{e}$ | 0.228$^{b}$ | 0.282$^{e}$ | 0.194$^{d}$ |
| | RSD | 0.668 | 0.916 | 0.642 | 1.646 | 2.178 | 1.592 | 1.512 | 1.176 |
| C16:1 pal-mitoleic acid | avg | 0.670 | 0.630 | 0.230 | 0.750 | 0.740 | 0.330 | 0.560 | 0.420 |
| | sd | 0.018$^{j}$ | 0.011$^{i}$ | 0.001$^{a}$ | 0.020$^{k}$ | 0.028$^{k}$ | 0.001$^{d}$ | 0.007$^{h}$ | 0.005$^{e}$ |
| | RSD | 2.672 | 1.802 | 0.504 | 2.712 | 3.770 | 0.266 | 1.316 | 1.304 |
| C17:0 margaric acid | avg | 0.480 | 0.420 | 0.210 | 0.560 | 0.590 | 0.220 | 0.520 | 0.420 |
| | sd | 0.004$^{e}$ | 0.005$^{d}$ | 0.004$^{a}$ | 0.010$^{g}$ | 0.012$^{h}$ | 0.004$^{a}$ | 0.010$^{f}$ | 0.006$^{d}$ |
| | RSD | 0.778 | 1.306 | 1.972 | 1.770 | 2.070 | 1.812 | 1.882 | 1.436 |
| C17:1 hep-tadesenoic acid | avg | 0.160 | 0.150 | 0.040 | 0.110 | 0.110 | 0.030 | 0.210 | 0.110 |
| | sd | 0.003$^{h}$ | 0.004$^{g}$ | 0.001$^{c}$ | 0.003$^{d}$ | 0.004$^{d}$ | 0.001$^{b}$ | 0.001$^{j}$ | 0.000$^{d}$ |
| | RSD | 1.856 | 2.957 | 2.152 | 3.012 | 3.995 | 3.442 | 0.289 | 0.258 |
| C18:0 stearic acid | avg | 6.580 | 6.960 | 3.120 | 7.560 | 7.990 | 4.110 | 5.630 | 4.250 |
| | sd | 0.199$^{ef}$ | 0.301$^{efg}$ | 0.094$^{b}$ | 0.260$^{hi}$ | 0.380$^{i}$ | 0.205$^{c}$ | 0.111$^{d}$ | 0.086$^{c}$ |
| | RSD | 3.018 | 4.325 | 2.998 | 3.441 | 4.756 | 4.998 | 1.963 | 2.022 |
| C18:1 trans-oleic acid | avg | 0.230 | 0.220 | 0.100 | 0.190 | 0.210 | 0.110 | 0.230 | 0.110 |
| | sd | 0.003$^{h}$ | 0.003$^{gh}$ | 0.001$^{bc}$ | 0.010$^{f}$ | 0.009$^{g}$ | 0.002$^{c}$ | 0.005$^{h}$ | 0.004$^{c}$ |
| | RSD | 1.325 | 1.503 | 1.145 | 5.112 | 4.245 | 2.268 | 2.145 | 3.689 |
| C18:1 cis-oleic acid | avg | 52.320 | 55.630 | 12.360 | 38.250 | 34.520 | 14.220 | 25.630 | 12.360 |
| | sd | 0.583$^{h}$ | 0.791$^{i}$ | 0.134$^{a}$ | 0.989$^{ef}$ | 1.495$^{d}$ | 0.315$^{a}$ | 0.425$^{c}$ | 0.122$^{a}$ |
| | RSD | 1.114 | 1.423 | 1.084 | 2.585 | 4.332 | 2.214 | 1.658 | 0.986 |
| C18:2 trans-lino-leic acid | avg | 0.070 | 0.070 | 0.020 | 0.080 | 0.060 | 0.040 | 0.060 | 0.050 |
| | sd | 0.004$^{f}$ | 0.003$^{f}$ | 0.000$^{b}$ | 0.002$^{g}$ | 0.002$^{e}$ | 0.000$^{c}$ | 0.001$^{e}$ | 0.001$^{d}$ |
| | RSD | 5.623 | 4.112 | 1.542 | 2.774 | 3.235 | 1.112 | 2.336 | 2.114 |
| C18:2 cis-linoleic acid | avg | 0.290 | 0.280 | 0.150 | 0.190 | 0.190 | 0.090 | 0.150 | 0.110 |
| | sd | 0.004$^{ab}$ | 0.003$^{ab}$ | 0.001$^{ab}$ | 0.002$^{ab}$ | 0.001$^{ab}$ | 0.002$^{a}$ | 0.001$^{ab}$ | 0.001$^{a}$ |
| | RSD | 1.021 | 2.356 | 2.985 | 2.114 | 1.985 | 1.665 | 2.883 | 2.898 |
| C18:3 linolenic acid | avg | 0.240 | 0.230 | 0.120 | 0.180 | 0.190 | 0.080 | 0.160 | 0.110 |
| | sd | 0.006$^{g}$ | 0.005$^{g}$ | 0.004$^{d}$ | 0.006$^{f}$ | 0.009$^{f}$ | 0.005$^{bc}$ | 0.008$^{e}$ | 0.007$^{d}$ |
| | RSD | 2.412 | 2.125 | 2.983 | 3.224 | 4.863 | 5.635 | 4.863 | 6.042 |
| C18:3 gamma | avg | 1.040 | 1.090 | 0.520 | 0.650 | 0.680 | 0.230 | 0.260 | 0.150 |

| | | | | | | | | | |
|---|---|---|---|---|---|---|---|---|---|
| | sd | 0.021$^{hi}$ | 0.036$^{i}$ | 0.022$^{e}$ | 0.013$^{f}$ | 0.024$^{f}$ | 0.010$^{c}$ | 0.008$^{cd}$ | 0.008$^{b}$ |
| | RSD | 2.035 | 3.325 | 4.325 | 1.996 | 3.487 | 4.235 | 3.025 | 5.023 |
| C20:0 arachidic acid | avg | 0.350 | 0.380 | 0.210 | 0.540 | 0.560 | 0.240 | 0.230 | 0.150 |
| | sd | 0.008$^{h}$ | 0.013$^{h}$ | 0.003$^{f}$ | 0.022$^{i}$ | 0.035$^{i}$ | 0.011$^{fg}$ | 0.004$^{fg}$ | 0.005$^{de}$ |
| | RSD | 2.252 | 3.356 | 1.332 | 4.021 | 6.258 | 4.563 | 1.853 | 3.124 |
| C20:1 eicosenoic acid | avg | 0.170 | 0.190 | 0.140 | 0.160 | 0.120 | 0.090 | 0.230 | 0.110 |
| | sd | 0.003$^{e}$ | 0.010$^{f}$ | 0.005$^{d}$ | 0.007$^{e}$ | 0.003$^{c}$ | 0.002$^{b}$ | 0.008$^{g}$ | 0.002$^{c}$ |
| | RSD | 1.896 | 5.423 | 3.321 | 4.658 | 2.332 | 1.996 | 3.562 | 2.112 |
| C20:2 eicosadienoic acid | avg | 0.120 | 0.150 | 0.110 | 0.210 | 0.150 | 0.070 | 0.150 | 0.120 |
| | sd | 0.004$^{c}$ | 0.006$^{d}$ | 0.004$^{c}$ | 0.004$^{e}$ | 0.003$^{d}$ | 0.001$^{b}$ | 0.003$^{d}$ | 0.003$^{c}$ |
| | RSD | 3.658 | 4.321 | 3.323 | 2.142 | 1.785 | 2.036 | 2.065 | 2.498 |
| C21:0 heneicosanoic acid | avg | 0.130 | 0.110 | 0.070 | 0.120 | 0.110 | 0.050 | 0.120 | 0.090 |
| | sd | 0.003$^{g}$ | 0.003$^{e}$ | 0.002$^{c}$ | 0.000$^{f}$ | 0.001$^{e}$ | 0.000$^{b}$ | 0.000$^{f}$ | 0.000$^{d}$ |
| | RSD | 2.256 | 2.853 | 2.996 | 0.285 | 0.998 | 0.743 | 0.233 | 0.284 |
| C20:3 $n$ = 3 cis-11,14,17-eicosatrienoic acid | avg | 0.110 | 0.090 | 0.030 | 0.130 | 0.080 | 0.020 | 0.110 | 0.060 |
| | sd | 0.000$^{g}$ | 0.000$^{f}$ | 0.000$^{b}$ | 0.001$^{h}$ | 0.001$^{e}$ | 0.000$^{a}$ | 0.000$^{g}$ | 0.000$^{c}$ |
| | RSD | 0.314 | 0.398 | 0.521 | 0.511 | 0.756 | 0.488 | 0.156 | 0.175 |
| C20:4 arachidonic acid | avg | 0.180 | 0.150 | 0.080 | 0.090 | 0.070 | 0.040 | 0.150 | 0.080 |
| | sd | 0.001$^{i}$ | 0.000$^{h}$ | 0.000$^{e}$ | 0.000$^{f}$ | 0.000$^{d}$ | 0.000$^{b}$ | 0.000$^{h}$ | 0.006$^{e}$ |
| | RSD | 0.285 | 0.255 | 0.506 | 0.489 | 0.110 | 0.075 | 0.296 | 7.226 |
| C20:3 $n$ = 6 cis-8,11,14-eicosatrienoic acid | avg | 0.260 | 0.210 | 0.040 | 0.050 | 0.030 | 0.010 | 0.130 | 0.090 |
| | sd | 0.001$^{l}$ | 0.001$^{k}$ | 0.000$^{d}$ | 0.000$^{e}$ | 0.000$^{c}$ | 0.000$^{a}$ | 0.001$^{j}$ | 0.000$^{h}$ |
| | RSD | 0.511 | 0.281 | 0.331 | 0.268 | 0.235 | 0.356 | 0.489 | 0.165 |
| C22:0 behenic acid | avg | 0.430 | 0.450 | 0.210 | 0.390 | 0.430 | 0.250 | 0.530 | 0.390 |
| | sd | 0.014$^{g}$ | 0.018$^{h}$ | 0.008$^{b}$ | 0.001$^{f}$ | 0.001$^{g}$ | 0.000$^{c}$ | 0.003$^{j}$ | 0.001$^{f}$ |
| | RSD | 3.336 | 3.983 | 4.014 | 0.189 | 0.203 | 0.188 | 0.523 | 0.195 |
| C20:5 eicosapentaenoic acid | avg | 0.230 | 0.210 | 0.110 | 0.320 | 0.240 | 0.150 | 0.180 | 0.110 |
| | sd | 0.001$^{i}$ | 0.000$^{g}$ | 0.000$^{b}$ | 0.001$^{k}$ | 0.001$^{j}$ | 0.001$^{d}$ | 0.001$^{e}$ | 0.001$^{b}$ |
| | RSD | 0.245 | 0.223 | 0.325 | 0.332 | 0.425 | 0.665 | 0.283 | 1.245 |
| C22:1 erucic acid | avg | 0.190 | 0.170 | 0.100 | 0.310 | 0.250 | 0.190 | 0.160 | 0.090 |
| | sd | 0.002$^{j}$ | 0.003$^{i}$ | 0.001$^{d}$ | 0.003$^{l}$ | 0.006$^{k}$ | 0.004$^{j}$ | 0.002$^{h}$ | 0.001$^{c}$ |
| | RSD | 1.104 | 1.548 | 1.442 | 1.124 | 2.326 | 1.886 | 1.083 | 1.653 |
| C22:2 docosadienoic acid | avg | 0.530 | 0.480 | 0.360 | 0.480 | 0.190 | 0.330 | 0.250 | 0.140 |
| | sd | 0.005$^{j}$ | 0.010$^{i}$ | 0.004$^{h}$ | 0.008$^{i}$ | 0.005$^{c}$ | 0.010$^{g}$ | 0.000$^{e}$ | 0.000$^{b}$ |
| | RSD | 1.021 | 2.032 | 0.996 | 1.563 | 2.441 | 2.896 | 0.189 | 0.252 |
| C23:0 tricosanoic acid | avg | 0.560 | 0.510 | 0.310 | 0.560 | 0.470 | 0.350 | 0.360 | 0.250 |
| | sd | 0.013$^{l}$ | 0.004$^{k}$ | 0.002$^{e}$ | 0.006$^{l}$ | 0.005$^{j}$ | 0.007$^{gh}$ | 0.006$^{h}$ | 0.008$^{bc}$ |
| | RSD | 2.331 | 0.752 | 0.552 | 1.025 | 0.998 | 1.885 | 1.589 | 3.057 |
| C24:0 lignoseric acid | avg | 0.420 | 0.440 | 0.210 | 0.590 | 0.510 | 0.330 | 0.420 | 0.360 |
| | sd | 0.002$^{g}$ | 0.002$^{h}$ | 0.001$^{a}$ | 0.002$^{j}$ | 0.002$^{i}$ | 0.001$^{d}$ | 0.008$^{g}$ | 0.004$^{f}$ |
| | RSD | 0.452 | 0.568 | 0.402 | 0.352 | 0.458 | 0.336 | 1.986 | 1.112 |
| C22:6 docosahexaenoic acid | avg | 0.510 | 0.530 | 0.390 | 0.610 | 0.580 | 0.430 | 0.590 | 0.410 |
| | sd | 0.005$^{g}$ | 0.004$^{h}$ | 0.003$^{d}$ | 0.004$^{k}$ | 0.006$^{j}$ | 0.004$^{f}$ | 0.007$^{j}$ | 0.004$^{e}$ |
| | RSD | 0.989 | 0.711 | 0.893 | 0.689 | 0.998 | 1.001 | 1.114 | 1.012 |

Legend: The results are expressed as mean values of three replicates ± standard deviation. RSD (relative standard deviation). Different letters in the same row indicate statistically significant difference at $p < 0.05$. Abbreviations: IS - Serbian Chaga, MW 96% EtOH, 50% EtOH, $H_2O$ - microwave-assisted extraction, VAE 96% EtOH, 50% EtOH, $H_2O$ - ultrasound-assisted extraction, SWE 200 °C, 120 °C.

**Table S6.** Fatty acid profile of Mongolian *I. obliquus* extracts obtained by different green extraction techniques mg/100 g.

| **mg/100 g** | | **IM-MW 96%ETOH** | **IM-MW 50% ETOH** | **IM-MW water** | **IM-VAE 96% ETOH** | **IM-VAE 50% ETOH** | **IM-VAE water** | **IM-SWE 200°C** | **IM-SWE 120°C** |
|---|---|---|---|---|---|---|---|---|---|
| C4:0 butyric acid | avg | 0.090 | 0.110 | 0.060 | 0.050 | 0.050 | 0.030 | 0.110 | 0.100 |
| | sd | $0.002^{g}$ | $0.001^{i}$ | $0.001^{e}$ | $0.001^{d}$ | $0.002^{d}$ | $0.001^{b}$ | $0.001^{i}$ | $0.001^{h}$ |
| | RSD | 1.999 | 0.666 | 0.998 | 2.845 | 3.162 | 3.668 | 1.239 | 0.968 |
| C6:0 caproic acid | avg | 0.080 | 0.070 | 0.050 | 0.090 | 0.080 | 0.040 | 0.110 | 0.080 |
| | sd | $0.001^{g}$ | $0.001^{f}$ | $0.001^{e}$ | $0.001^{h}$ | $0.002^{g}$ | $0.001^{d}$ | $0.002^{i}$ | $0.001^{g}$ |
| | RSD | 1.345 | 1.228 | 1.493 | 1.371 | 1.896 | 2.331 | 1.852 | 1.452 |
| C8:0 caprilic acid | avg | 0.150 | 0.130 | 0.080 | 0.130 | 0.110 | 0.080 | 0.150 | 0.180 |
| | sd | $0.001^{h}$ | $0.004^{g}$ | $0.001^{d}$ | $0.006^{g}$ | $0.005^{f}$ | $0.004^{d}$ | $0.001^{h}$ | $0.006^{k}$ |
| | RSD | 0.920 | 3.194 | 0.948 | 4.899 | 4.999 | 5.426 | 0.568 | 3.326 |
| C10:0 capric acid | avg | 0.480 | 0.490 | 0.240 | 0.330 | 0.360 | 0.140 | 0.630 | 0.520 |
| | sd | $0.019^{f}$ | $0.023^{f}$ | $0.007^{c}$ | $0.001^{d}$ | $0.002^{d}$ | $0.001^{a}$ | $0.009^{h}$ | $0.012^{f}$ |
| | RSD | 3.936 | 4.778 | 2.965 | 0.366 | 0.457 | 0.386 | 1.395 | 2.281 |
| C11:0 undeca-noic acid | avg | 0.210 | 0.230 | 0.110 | 0.250 | 0.210 | 0.110 | 0.530 | 0.410 |
| | sd | $0.006^{d}$ | $0.003^{de}$ | $0.002^{b}$ | $0.004^{e}$ | $0.006^{d}$ | $0.002^{b}$ | $0.012^{h}$ | $0.005^{g}$ |
| | RSD | 2.816 | 1.423 | 1.950 | 1.486 | 2.708 | 2.185 | 2.255 | 1.299 |
| C12:0 lauric acid | avg | 0.180 | 0.170 | 0.070 | 0.230 | 0.220 | 0.080 | 0.330 | 0.210 |
| | sd | $0.008^{fg}$ | $0.009^{f}$ | $0.003^{bc}$ | $0.007^{i}$ | $0.006^{hi}$ | $0.000^{c}$ | $0.015^{j}$ | $0.011^{h}$ |
| | RSD | 4.432 | 5.312 | 4.278 | 2.979 | 2.697 | 0.044 | 4.568 | 5.128 |
| C13:0 tride-canoic acid | avg | 0.060 | 0.070 | 0.030 | 0.080 | 0.080 | 0.400 | 0.130 | 0.110 |
| | sd | $0.000^{de}$ | $0.002^{ef}$ | $0.001^{ab}$ | $0.002^{f}$ | $0.003^{f}$ | $0.013^{i}$ | $0.002^{h}$ | $0.001^{g}$ |
| | RSD | 0.519 | 2.258 | 3.056 | 3.035 | 3.580 | 3.266 | 1.320 | 1.242 |
| C14:0 myristic acid | avg | 0.810 | 0.830 | 0.320 | 0.720 | 0.750 | 0.410 | 0.840 | 0.520 |
| | sd | $0.021^{j}$ | $0.012^{j}$ | $0.006^{b}$ | $0.012^{gh}$ | $0.012^{h}$ | $0.008^{c}$ | $0.022^{j}$ | $0.016^{d}$ |
| | RSD | 2.556 | 1.492 | 1.775 | 1.623 | 1.542 | 1.985 | 2.568 | 3.124 |
| C14:1 myristoleic acid | avg | 0.480 | 0.440 | 0.210 | 0.520 | 0.530 | 0.220 | 0.670 | 0.320 |
| | sd | $0.007^{e}$ | $0.017^{d}$ | $0.003^{a}$ | $0.007^{f}$ | $0.008^{f}$ | $0.004^{a}$ | $0.008^{i}$ | $0.003^{b}$ |
| | RSD | 1.522 | 3.790 | 1.316 | 1.424 | 1.578 | 1.624 | 1.266 | 0.796 |
| C15:0 pentadeca-noic acid | avg | 0.990 | 1.010 | 0.330 | 1.150 | 1.080 | 0.520 | 1.660 | 1.110 |
| | sd | $0.012^{ef}$ | $0.016^{fg}$ | $0.004^{a}$ | $0.005^{i}$ | $0.007^{h}$ | $0.003^{c}$ | $0.008^{l}$ | $0.012^{h}$ |
| | RSD | 1.226 | 1.622 | 1.104 | 0.464 | 0.622 | 0.596 | 0.470 | 1.072 |
| C15:1 pen-tadesenoic acid | avg | 0.080 | 0.070 | 0.020 | 0.090 | 0.060 | 0.020 | 0.140 | 0.090 |
| | sd | $0.001^{g}$ | $0.001^{f}$ | $0.000^{b}$ | $0.001^{h}$ | $0.000^{e}$ | $0.000^{b}$ | $0.001^{j}$ | $0.001^{h}$ |
| | RSD | 0.756 | 1.316 | 1.792 | 1.224 | 0.650 | 0.258 | 1.046 | 0.904 |
| C16:0 palmitic acid | avg | 14.630 | 13.890 | 10.320 | 15.630 | 16.010 | 10.630 | 21.350 | 18.630 |
| | sd | $0.231^{bc}$ | $0.293^{b}$ | $0.153^{a}$ | $0.059^{cd}$ | $0.052^{d}$ | $0.139^{a}$ | $0.800^{e}$ | $0.869^{e}$ |
| | RSD | 1.578 | 2.112 | 1.482 | 0.378 | 0.326 | 1.306 | 3.748 | 4.664 |
| C16:1 pal-mitoleic acid | avg | 0.530 | 0.490 | 0.210 | 0.280 | 0.290 | 0.180 | 0.630 | 0.510 |
| | sd | $0.001^{gh}$ | $0.007^{f}$ | $0.004^{ab}$ | $0.002^{c}$ | $0.003^{c}$ | $0.002^{a}$ | $0.012^{i}$ | $0.010^{fg}$ |
| | RSD | 0.272 | 1.430 | 1.748 | 0.824 | 1.046 | 1.094 | 1.926 | 1.906 |
| C17:0 margaric acid | avg | 0.620 | 0.650 | 0.250 | 0.710 | 0.760 | 0.320 | 0.680 | 0.520 |
| | sd | $0.004^{i}$ | $0.005^{j}$ | $0.002^{b}$ | $0.005^{l}$ | $0.010^{m}$ | $0.007^{c}$ | $0.009^{k}$ | $0.010^{f}$ |
| | RSD | 0.712 | 0.698 | 0.846 | 0.730 | 1.296 | 2.110 | 1.308 | 1.982 |
| | avg | 0.160 | 0.140 | 0.030 | 0.110 | 0.110 | 0.020 | 0.190 | 0.130 |

| | | | | | | | | | |
|---|---|---|---|---|---|---|---|---|---|
| C17:1 heptadesenoic acid | sd | 0.006[h] | 0.002[f] | 0.001[b] | 0.003[d] | 0.003[d] | 0.001[a] | 0.000[i] | 0.003[e] |
| | RSD | 3.562 | 1.553 | 2.856 | 2.994 | 2.452 | 3.325 | 0.241 | 2.141 |
| C18:0 stearic acid | avg | 6.420 | 6.56 | 3.880 | 7.010 | 7.230 | 4.110 | 2.530 | 2.110 |
| | sd | 0.227[e] | 0.156[ef] | 0.121[c] | 0.130[fgh] | 0.161[gh] | 0.084[c] | 0.045[a] | 0.068[a] |
| | RSD | 3.542 | 2.371 | 3.114 | 1.852 | 2.231 | 2.035 | 1.785 | 3.221 |
| C18:1 trans-oleic acid | avg | 0.180 | 0.190 | 0.090 | 0.150 | 0.130 | 0.040 | 0.250 | 0.160 |
| | sd | 0.004[f] | 0.004[f] | 0.003[b] | 0.003[e] | 0.002[d] | 0.000[a] | 0.005[i] | 0.002[e] |
| | RSD | 2.310 | 2.289 | 2.986 | 2.199 | 1.422 | 0.785 | 2.002 | 1.458 |
| C18:1 cis-oleic acid | avg | 48.630 | 49.210 | 21.330 | 36.520 | 39.780 | 14.330 | 35.850 | 12.230 |
| | sd | 1.408[g] | 1.636[g] | 0.523[b] | 1.676[de] | 1.295[f] | 0.159[a] | 0.881[de] | 0.381[a] |
| | RSD | 2.896 | 3.325 | 2.452 | 4.589 | 3.256 | 1.112 | 2.458 | 3.114 |
| C18:2 trans-linoleic acid | avg | 0.070 | 0.060 | 0.020 | 0.080 | 0.040 | 0.020 | 0.010 | 0.010 |
| | sd | 0.001[f] | 0.001[e] | 0.000[b] | 0.002[g] | 0.001[c] | 0.000[b] | 0.000[a] | 0.000[a] |
| | RSD | 1.085 | 0.989 | 1.254 | 2.117 | 3.256 | 1.985 | 1.236 | 1.023 |
| C18:2 cis-linoleic acid | avg | 1.109 | 1.526 | 1.996 | 0.223 | 0.356 | 0.452 | 0.388 | 0.345 |
| | sd | 0.210[c] | 0.230[d] | 0.090[e] | 0.150[ab] | 0.160[ab] | 0.080[b] | 0.100[ab] | 0.090[ab] |
| | RSD | 2.852 | 2.481 | 3.256 | 2.856 | 2.542 | 3.952 | 3.256 | 2.921 |
| C18:3 linolenic acid | avg | 0.180 | 0.160 | 0.080 | 0.090 | 0.080 | 0.020 | 0.120 | 0.070 |
| | sd | 0.009[f] | 0.008[e] | 0.005[bc] | 0.002[c] | 0.004[bc] | 0.001[a] | 0.011[d] | 0.006[b] |
| | RSD | 5.114 | 5.032 | 6.521 | 2.021 | 5.003 | 7.065 | 8.768 | 9.032 |
| C18:3 gamma | avg | 0.990 | 0.850 | 0.140 | 0.120 | 0.150 | 0.050 | 0.310 | 0.140 |
| | sd | 0.032[h] | 0.009[g] | 0.003[b] | 0.002[b] | 0.002[b] | 0.002[a] | 0.014[d] | 0.006[b] |
| | RSD | 3.225 | 1.023 | 1.856 | 1.562 | 1.335 | 3.253 | 4.633 | 4.589 |
| C20:0 arachidic acid | avg | 0.150 | 0.160 | 0.080 | 0.130 | 0.110 | 0.060 | 0.260 | 0.120 |
| | sd | 0.003[de] | 0.003[e] | 0.001[ab] | 0.008[cde] | 0.003[bc] | 0.002[a] | 0.004[g] | 0.004[cd] |
| | RSD | 2.325 | 1.856 | 1.221 | 6.214 | 2.956 | 3.889 | 1.623 | 3.224 |
| C20:1 eicosenoic acid | avg | 0.110 | 0.120 | 0.040 | 0.080 | 0.080 | 0.050 | 0.230 | 0.140 |
| | sd | 0.003[c] | 0.004[c] | 0.001[a] | 0.002[b] | 0.003[b] | 0.002[a] | 0.005[g] | 0.003[d] |
| | RSD | 2.358 | 3.124 | 2.258 | 2.998 | 3.385 | 3.118 | 2.325 | 1.996 |
| C20:2 eicosadienoic acid | avg | 0.080 | 0.070 | 0.050 | 0.110 | 0.120 | 0.080 | 0.180 | 0.140 |
| | sd | 0.001[b] | 0.001[b] | 0.001[a] | 0.004[c] | 0.004[c] | 0.002[b] | 0.004[e] | 0.005[d] |
| | RSD | 1.089 | 0.996 | 2.325 | 3.326 | 2.998 | 2.323 | 2.331 | 3.332 |
| C21:0 heneicosanoic acid | avg | 0.130 | 0.150 | 0.070 | 0.090 | 0.090 | 0.030 | 0.140 | 0.090 |
| | sd | 0.001[g] | 0.001[i] | 0.001[c] | 0.000[d] | 0.001[d] | 0.000[a] | 0.000[h] | 0.000[d] |
| | RSD | 0.712 | 0.963 | 0.822 | 0.426 | 0.689 | 0.411 | 0.314 | 0.114 |
| C20:3 $n$ = 3 cis-11,14,17-eicosatrienoic acid | avg | 0.140 | 0.140 | 0.080 | 0.060 | 0.070 | 0.020 | 0.150 | 0.020 |
| | sd | 0.001[i] | 0.001[i] | 0.000[e] | 0.000[c] | 0.000[d] | 0.000[a] | 0.000[j] | 0.000[a] |
| | RSD | 0.433 | 0.568 | 0.612 | 0.441 | 0.562 | 0.411 | 0.163 | 0.085 |
| C20:4 arachidonic acid | avg | 0.060 | 0.040 | 0.030 | 0.070 | 0.070 | 0.030 | 0.130 | 0.090 |
| | sd | 0.000[c] | 0.000[b] | 0.000[a] | 0.000[d] | 0.000[d] | 0.000[a] | 0.005[g] | 0.000[f] |
| | RSD | 0.163 | 0.141 | 0.285 | 0.293 | 0.288 | 0.355 | 3.526 | 0.523 |
| C20:3 $n$ = 6 cis-8,11,14-eicosatrienoic acid | avg | 0.060 | 0.060 | 0.010 | 0.050 | 0.050 | 0.020 | 0.110 | 0.070 |
| | sd | 0.000[f] | 0.000[f] | 0.000[a] | 0.000[e] | 0.000[e] | 0.000[b] | 0.000[i] | 0.000[g] |
| | RSD | 0.204 | 0.432 | 0.568 | 0.399 | 0.523 | 0.632 | 0.174 | 0.235 |
| C22:0 behenic acid | avg | 0.360 | 0.900 | 0.150 | 0.320 | 0.360 | 0.210 | 0.480 | 0.210 |
| | sd | 0.001[f] | 0.005[k] | 0.001[a] | 0.003[d] | 0.004[f] | 0.001[b] | 0.001[i] | 0.001[b] |
| | RSD | 0.332 | 0.568 | 0.865 | 0.998 | 1.024 | 0.487 | 0.206 | 0.336 |
| C20:5 eicosapentaenoic acid | avg | 0.190 | 0.140 | 0.080 | 0.230 | 0.220 | 0.110 | 0.220 | 0.150 |
| | sd | 0.001[f] | 0.000[c] | 0.000[a] | 0.001[i] | 0.001[h] | 0.000[b] | 0.006[h] | 0.003[d] |

| | | | | | | | | | |
|---|---|---|---|---|---|---|---|---|---|
| | RSD | 0.542 | 0.213 | 0.135 | 0.356 | 0.331 | 0.368 | 2.896 | 2.114 |
| C22:1 erucic acid | avg | 0.150 | 0.120 | 0.070 | 0.100 | 0.090 | 0.060 | 0.160 | 0.110 |
| | sd | 0.001$^{g}$ | 0.002$^{f}$ | 0.001$^{b}$ | 0.001$^{d}$ | 0.001$^{c}$ | 0.001$^{a}$ | 0.002$^{h}$ | 0.002$^{e}$ |
| | RSD | 0.993 | 1.856 | 0.874 | 1.125 | 1.563 | 1.983 | 1.112 | 1.941 |
| C22:2 docosadienoic acid | avg | 0.250 | 0.210 | 0.180 | 0.190 | 0.150 | 0.110 | 0.290 | 0.210 |
| | sd | 0.001$^{e}$ | 0.002$^{d}$ | 0.001$^{c}$ | 0.001$^{c}$ | 0.002$^{b}$ | 0.001$^{a}$ | 0.001$^{f}$ | 0.002$^{d}$ |
| | RSD | 0.452 | 0.883 | 0.398 | 0.623 | 1.124 | 0.853 | 0.177 | 1.022 |
| C23:0 tricosanoic acid | avg | 0.290 | 0.260 | 0.230 | 0.420 | 0.340 | 0.260 | 0.330 | 0.240 |
| | sd | 0.005$^{d}$ | 0.003$^{c}$ | 0.005$^{a}$ | 0.005$^{i}$ | 0.002$^{fg}$ | 0.001$^{c}$ | 0.004$^{f}$ | 0.001$^{ab}$ |
| | RSD | 1.632 | 1.256 | 2.325 | 1.268 | 0.562 | 0.421 | 1.121 | 0.401 |
| C24:0 lignoseric acid | avg | 0.230 | 0.250 | 0.210 | 0.350 | 0.360 | 0.210 | 0.510 | 0.420 |
| | sd | 0.002$^{b}$ | 0.003$^{c}$ | 0.002$^{a}$ | 0.001$^{e}$ | 0.002$^{f}$ | 0.003$^{a}$ | 0.001$^{i}$ | 0.001$^{g}$ |
| | RSD | 0.993 | 1.256 | 0.945 | 0.423 | 0.554 | 1.325 | 0.258 | 0.347 |
| C22:6 docosahexaenoic acid | avg | 0.510 | 0.560 | 0.330 | 0.370 | 0.390 | 0.260 | 0.630 | 0.510 |
| | sd | 0.005$^{g}$ | 0.003$^{i}$ | 0.002$^{b}$ | 0.003$^{c}$ | 0.003$^{d}$ | 0.002$^{a}$ | 0.004$^{l}$ | 0.003$^{g}$ |
| | RSD | 0.997 | 0.604 | 0.689 | 0.699 | 0.741 | 0.883 | 0.652 | 0.526 |

Legend: The results are expressed as mean values of three replicates ± standard deviation. RSD (relative standard deviation). Different letters in the same row indicate statistically significant difference at $p < 0.05$. Abbreviations: IS - Serbian Chaga, MW 96% EtOH, 50% EtOH, $H_2O$ - microwave-assisted extraction, VAE 96% EtOH, 50% EtOH, $H_2O$ - ultrasound-assisted extraction, SWE 200 °C, 120 °C.
